# Genomic Variability within an Organism Exposes Its Cell Lineage Tree

**DOI:** 10.1371/journal.pcbi.0010050

**Published:** 2005-10-28

**Authors:** Dan Frumkin, Adam Wasserstrom, Shai Kaplan, Uriel Feige, Ehud Shapiro

**Affiliations:** 1 Department of Biological Chemistry, Weizmann Institute of Science, Rehovot, Israel; 2 Department of Computer Science and Applied Mathematics, Weizmann Institute of Science, Rehovot, Israel; Swiss Federal Institute of Technology, Switzerland

## Abstract

What is the lineage relation among the cells of an organism? The answer is sought by developmental biology, immunology, stem cell research, brain research, and cancer research, yet complete cell lineage trees have been reconstructed only for simple organisms such as *Caenorhabditis elegans*. We discovered that somatic mutations accumulated during normal development of a higher organism implicitly encode its entire cell lineage tree with very high precision. Our mathematical analysis of known mutation rates in microsatellites (MSs) shows that the entire cell lineage tree of a human embryo, or a mouse, in which no cell is a descendent of more than 40 divisions, can be reconstructed from information on somatic MS mutations alone with no errors, with probability greater than 99.95%. Analyzing all ~1.5 million MSs of each cell of an organism may not be practical at present, but we also show that in a genetically unstable organism, analyzing only a few hundred MSs may suffice to reconstruct portions of its cell lineage tree. We demonstrate the utility of the approach by reconstructing cell lineage trees from DNA samples of a human cell line displaying MS instability. Our discovery and its associated procedure, which we have automated, may point the way to a future “Human Cell Lineage Project” that would aim to resolve fundamental open questions in biology and medicine by reconstructing ever larger portions of the human cell lineage tree.

## Synopsis

The human body is made of about 100 trillion cells, all of which are descendants of a single cell, the fertilized egg. The quest to understand their path of descent, called a cell lineage tree, is shared by many branches of biology and medicine, including developmental biology, immunology, stem cell research, brain research, and cancer research. So far, science has been able to determine the cell lineage tree of tiny organisms only, worms with a thousand cells or so. Our team has discovered that the mutations accumulated in each cell in our body during its normal development from the zygote carry sufficient information to reconstruct, in principle, cell lineage trees for large organisms, including humans. Inspired by this discovery, we developed an automated procedure for the reconstruction of cell lineage trees from DNA samples. A direct application of these results may include the analysis of the development of cancer. The results may also inspire a future “Human Cell Lineage Project,” whose aim would be to reconstruct an entire human cell lineage tree.

## Introduction

**Figure 1:**
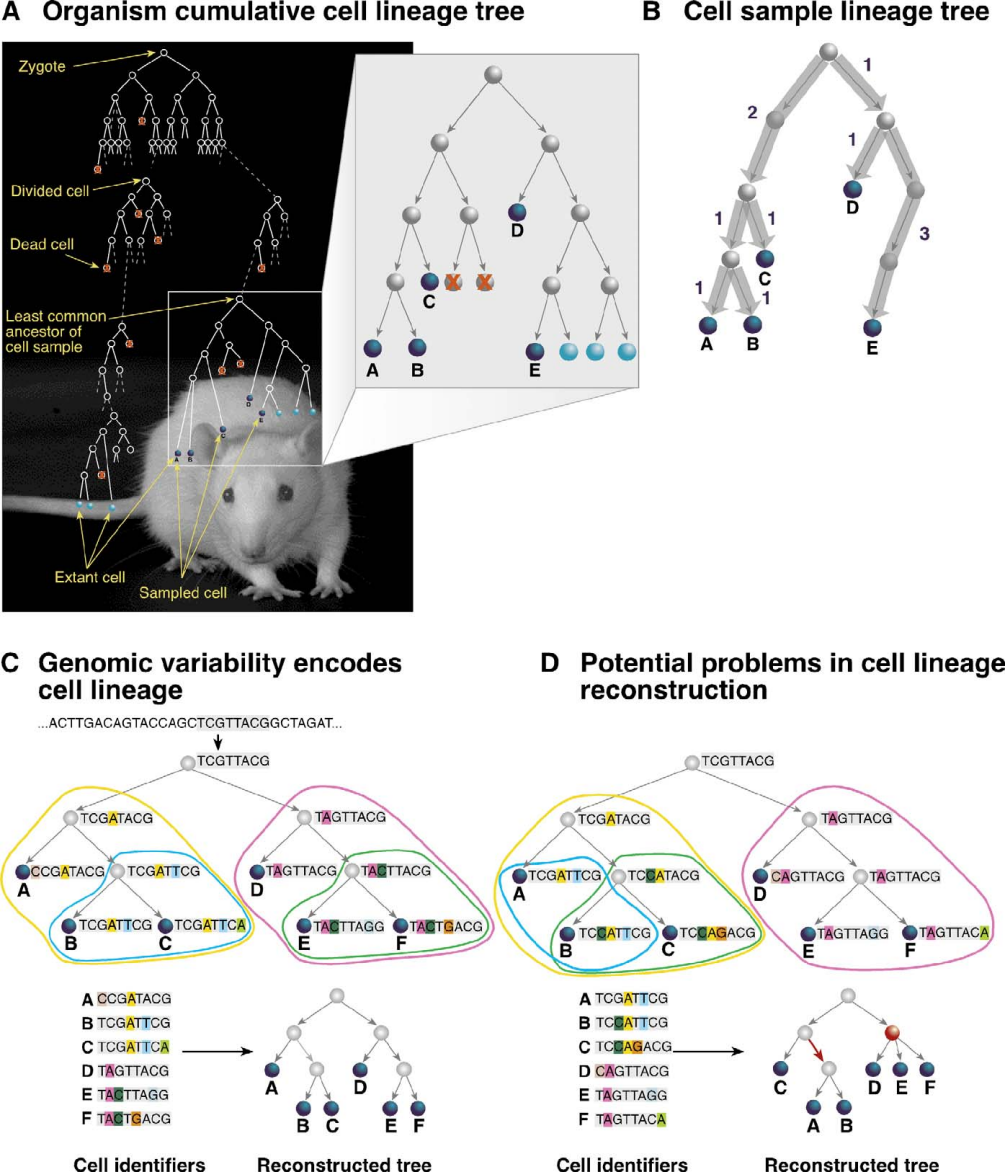
Cell Lineage Concepts (A) Multicellular organism development can be represented by a rooted labeled binary tree called the organism cumulative cell lineage tree. Nodes (circles) represent cells (dead cells are crossed), and each edge (line) connects a parent with a daughter. The uncrossed leaves, marked blue, represent extant cells. (B) Any cell sample (A–E) induces a subtree, which can be condensed by removing nonbranching internal nodes and labeling the edges with the number of cell divisions between the remaining nodes. The resulting tree is called the cell sample lineage tree. (C) A small fraction of a genome accumulating substitution mutations (colored) is shown. Lineage analysis utilizes a representation of this small fraction, called the cell identifier. Phylogenetic analysis reconstructs the tree from the cell identifiers of the samples. If the topology of the cell sample lineage tree is known, reconstruction can be scored. (D) Coincident mutations, namely two or more identical mutations that occur independently in different cell divisions (blue mutation in A and B), and silent cell divisions, namely cell divisions in which no mutation occurs (D–F), may result in incorrect (red edge) or incomplete (unresolved ternary red node) lineage trees. Excessive mutation rates might result in successive mutations (not shown), which cause the lineage information to be lost.

A multicellular organism develops from a single cell, the zygote, through numerous binary cell divisions and cell deaths. Consequently, at any given time, the lineage relations between the cells of the organism can be represented by a rooted labeled binary tree called the cumulative cell lineage tree (Figure 1A). For any sample of cells, such as cells from a specific organ or tissue, the lineage relations can also be represented by a tree, called the cell sample lineage tree, which is partial to the cumulative tree (Figure 1B). Such a tree was reconstructed for the 959 somatic cells of *Caenorhabditis elegans* by direct observation of cell divisions [1], a technique that can be used for lineage analysis of small transparent organisms. Understanding the cell lineage trees of higher organisms, especially human, is a fundamental challenge of many branches of biology [2–10] and medicine [11–15]. Development of higher organisms is, however, less deterministic than that of *C. elegans,* and therefore the cell lineage trees of individuals of the same species may vary considerably.

Lineage relations among cells have been studied using a variety of clonal assays [2,3,6,8,10,16–24]. Such assays act by detecting the progeny of a single founder cell, which has been marked by a heritable marker. Some assays mark the founder cell by an invasive technique such as injection of a tracer molecule [16,18] or retroviral infection [10], which may interfere with the normal growth and biological function of the marked cell population. Other noninvasive clonal assays are based on spontaneous mutations in the founder cell, for example, the loss or gain of large genomic fragments [19], mitochondrial DNA mutations [20], T-cell receptor gene recombination [21], and changes in the number of microsatellite (MS) repeat units [15,24]. Epigenetic changes have also been used for clonal assays [22] and for determining stem cell growth dynamics [23]. A clonal assay provides limited lineage information because it determines only whether certain cells are descendants of the founder cell.

Genetic variability has also been used for reconstructing lineage trees of several tissue samples extracted from the same individual. In one study [25], tissue samples from breast cancer patients were analyzed for loss of heterozygosity and mutations in mitochondrial DNA, and the result of this analysis was fed into a phylogenetic algorithm, yielding tissue lineage trees. In a different study [26], lineage trees of colorectal cancer and adenoma tissue samples were reconstructed from mutations in MS loci. These studies applied clustering algorithms to genetic variability among heterogeneous tissue samples. However, the meaning of the output of such an algorithmic analysis is not necessarily clear, as the lineage relations among tissue samples, where one or more tissue samples contain cells of heterogeneous lineage, are normally not amenable to simple representation via binary trees. On the other hand, the lineage relations among single cells or discrete cell clones can be represented naturally via rooted labeled binary trees, and the question of whether such a particular mathematical tree faithfully represents the lineage relations among a given set of cells or cell clones sampled from an individual is well-defined and has a simple yes/no answer (see Text S1). Genomic variability in immunoglobulin genes has also been used to create mutational lineage trees [27] in the study of the dynamics of selection in the immune system. Because this work analyzed mutations in a functional gene, which determines the ability of the cell to undergo clonal expansion, the shape of the mutational lineage trees reflects primarily selection forces and does not necessarily correlate to the cell lineage tree.

In this paper we show that cell lineage trees can be reconstructed from genomic variability caused by somatic mutations, and that somatic mutations in higher organisms contain sufficient information to allow precise reconstruction of the organism cell lineage tree. We describe a hybrid in vitro/in silico automated procedure for reconstructing cell lineage trees from DNA samples, and demonstrate its effectiveness and precision in a controlled environment.

## Results

### Somatic Genomic Variability Encodes Cell Lineage

Somatic mutations are sufficiently rare for common wisdom to say that “the genome is the same in every cell in the body” except for some white blood cells [28] and except for cancer [26]. We discovered that somatic mutations accumulated during normal development of a higher organism, including human and mouse, implicitly encode its entire cell lineage tree with very high precision.

Figure 1C shows how accumulated somatic mutations, each viewed as an implicit clonal marker, may encode a cell lineage tree. As we show, sufficiently many markers enable the inference of the cell lineage tree, as closely related cells tend to share more markers than distantly related cells. If in each cell division each daughter cell acquires a new mutation, and all mutations are unique and persistent, then the organism cell lineage tree can be precisely reconstructed from this mutation information, using known phylogenetic algorithms [29].

However, precise reconstruction may be hampered by three factors: coincident mutations (Figure 1D), silent cell divisions (Figure 1D), and successive mutations (see proof of theorem 1 in the Materials and Methods). Still, known phylogenetic algorithms can produce useful lineage information in spite of these problems if the mutations carry sufficient information with a sufficiently high “signal-to-noise” ratio. Although phylogenetic analysis algorithms were originally developed for reconstructing lineage trees of species [30], they are also applicable to cell lineage tree reconstruction. It is conceivable, though, that they would be outperformed by algorithms designed specifically for this new task. Such an algorithm may use a more accurate model of MS mutation behavior and make use of the precise root information, which may be obtained in organism cell lineage trees, in order to reconstruct the tree more accurately.

In principle, any mutation information may assist lineage tree reconstruction. We focus on mutations in MSs for the following reasons: (1) MS slippage mutations, which insert or delete repeated units in an MS, are thought to occur during DNA replication [31] and hence are coupled to cell division; (2) MS mutations occur at relatively high rates [31] and offer a broad range of rates to choose from; (3) MS mutations are believed to occur independently at different loci, usually without affecting phenotype, and are unlikely to be selected against somatically since most are found in noncoding genomic sequences; (4) MSs are highly abundant in human, mouse, and many other organisms [31]; and (5) animals with mutations in key mismatch repair (MMR) genes display very high mutation rates in MSs [32,33] in all tissues and are available for experimentation and analysis. MMR-deficient humans [32] and mice [33] have been shown to develop normally, albeit with a high incidence of cancer. Genetic variability of MS loci has been used for linkage analysis [34], individual identification [35], phylogenetic analysis of species [36], and genealogical history analysis of populations [37].

### Theoretical Potential of the Method

**Figure 2:**
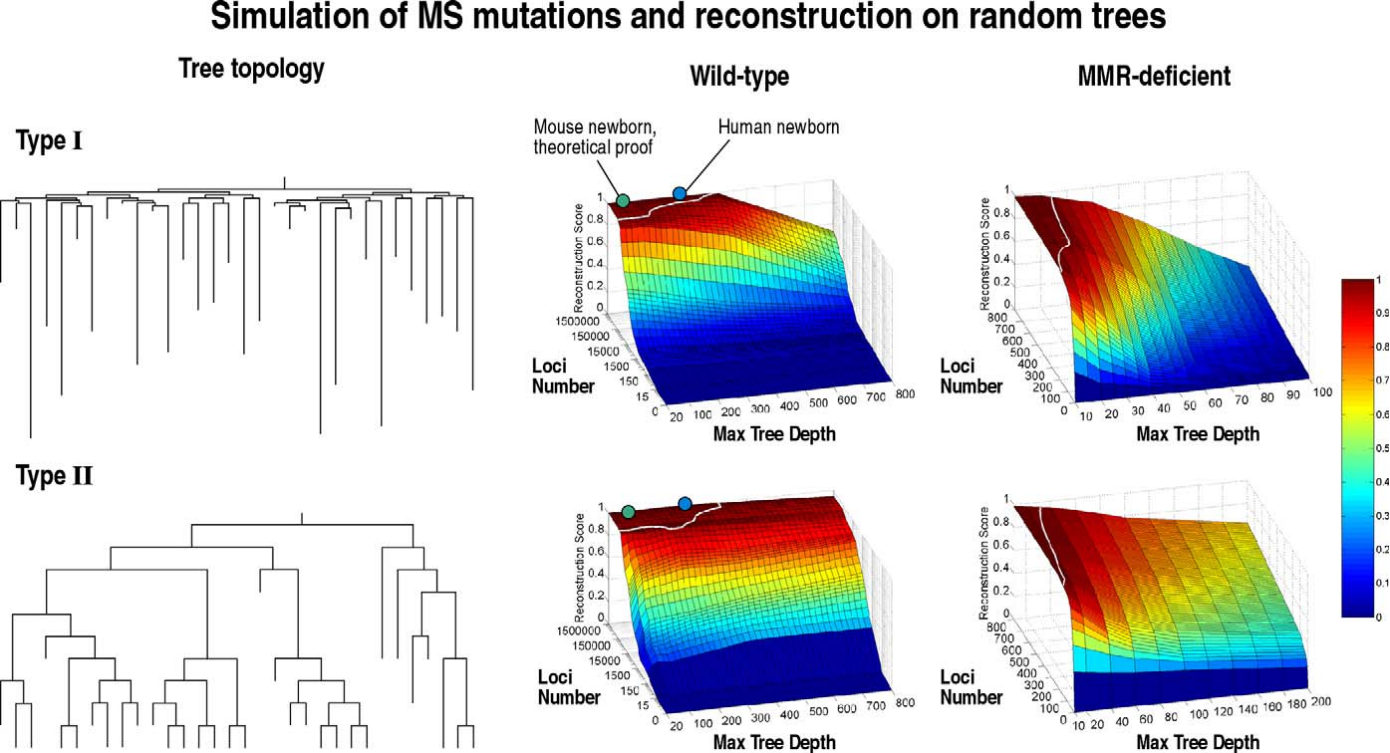
Simulation of MS Mutations and Reconstruction Score on Random Trees Two types of random trees with 32 leaves were generated, and MS stepwise mutations were simulated. Results of simulations of wild-type human using different numbers of MS loci are shown. The white line marks the perfect score limit (according to the Penny and Hendy tree comparison algorithm [29]). The results show that it is possible to accurately reconstruct the correct tree for trees of depth equivalent to human newborn and mouse newborn (marked by blue and green dots, respectively) using the entire set of MS loci. A mathematical analysis proves that any tree of depth 40 (equivalent to mouse newborn) can be reconstructed with no errors. Simulations with MS mutation rates of MMR-deficient organisms demonstrate that cell lineage reconstruction is possible with as few as 800 MS loci (the white line indicates the 0.95 score). The quality of reconstruction depends on the topology of the tree and its maximal depth, which together influence the signal-to-noise ratio.

In order to asses the theoretical potential of lineage analysis using genomic MSs, we obtained data regarding human and mouse MSs and performed calculations and computer simulations based on these data. We searched the human and mouse genomes for MSs and found about 1.5 million loci interspersed on all chromosomes and containing a variable number of tandem repeats. Based on this data, and on published data regarding human and mouse MS mutation rates [38], we calculated that in each cell division in wild-type humans and mice, each daughter cell acquires on average approximately 50 new mutations in MS loci. Based on this information, we were able to prove theorem 1 (see below), which implies that in human and mouse lineage trees with a maximum depth of 40 cell divisions (which can contain up to 10^12^ leaves and correspond, under reasonable assumptions, to a newborn mouse; Figure 2) and with any topology, the complete cell lineage tree can be reconstructed with no errors with a probability greater than 99.95%.

### The Possibility of Precise Reconstruction of Cell Lineage Trees Not Deeper Than 40 Cell Divisions

We prove that our approach has the potential of reconstructing without error condensed trees of sets of cells that are many orders of magnitude larger than anything achieved in the past. Our proof is based on certain assumptions regarding the DNA contents and nature of mutations in the human genome. These assumptions are stated explicitly in this manuscript, and (to the best of our knowledge) are in agreement with existing biological literature.

In describing our theoretical results, we prefer simplicity over achieving the best possible results based on our assumptions. In particular, the “triplet algorithm” that we present for reconstructing the condensed tree does not make use of all information available to it, and there is a lot of slack in the analysis. One may expect that more sophisticated algorithms coupled with tighter analysis will allow one to extend the family of trees that can be inferred using our methods.

We have chosen not to try to strengthen our theoretical results at this point for the following reasons. First, we believe that the vast potential of our method is made clear already by the analysis that we provide here. Second, and perhaps more importantly, it may be premature to enter into a lengthy theoretical analysis before establishing more firmly the biological assumptions on which the analysis is based. The biological assumptions that we make here may eventually turn out to be too optimistic in some respects (e.g., that mutation events are, or can be viewed as being, statistically independent), and too pessimistic in other respects (e.g., that the only significant source of variability in the human genome is MSs). Hence, there is not much point in performing tedious and time-consuming analysis based on current biological assumptions.

We now describe the mutation model that we assume in the analysis, which we call the “uniform model.” We use the following notation: *m,* number of MS loci; *p,* probability of mutation per MS locus per cell division; *d,* maximum depth of leaves in the cumulative tree; *n,* number of extant cells. The main assumption for the uniform model is that all mutation events are statistically independent. The simplifying assumptions (which simplify the presentation, but can be relaxed without qualitatively changing the results) are the following: (1) the identifier (a vector representing MS lengths) of the root is known and used as a reference; (2) both daughter cells of the root lead to extant cells; (3) all loci have the same mutation probability; and (4) mutations are stepwise, with equal probability for +1 and −1. The numerical values that are used in our analysis of the uniform model (roughly corresponding to known information about wild-type human MSs) are *m* = 2 × 10^6^ and *p* = 2.5 × 10^−5^.

This completes the description of the uniform model. Our analysis addresses the following question: assuming that one could read with no error the identifiers of all extant cells, can one (with high probability, over the events of random mutations) reconstruct the underlying condensed tree with no error at all? The answer depends on the “shape” of the extant tree. We present some ranges of parameters for which reconstruction is possible. Specifically, we take *d* = 40, and *n* as being arbitrary (but of course, no more than 2^40^). Note that *n* may be “in the same ball park” as the number of extant cells in a human (which is believed to be around 2^47^).

**Theorem 1:** Assuming the uniform model, with probability above 0.9995 (over the random mutation events), the genetic information in human cells suffices in order to reconstruct without error the condensed version of any extant tree of depth at most 40, regardless of the number of extant cells and the shape of the tree.

**Proof:** See Materials and Methods. It is not easy to extend this analytical result to trees deeper than 40 cell divisions; however, we have good reasons to believe that the theorem does not represent a singular data point, as shown by our computer simulations.

### Computer Simulations

We performed simulations on two types of randomly generated cell lineage trees (Figure 2), and simulated wild-type and MMR-deficient mutational behavior on hypothetical organisms that develop according to the pattern of these trees. The topology of cell sample lineage trees with 32 randomly chosen cells was then reconstructed based on analysis of mutations in a set of MSs. In wild-type human and mouse, using the entire set of genomic MSs yields accurate reconstruction in trees with a depth of several hundred cell divisions, corresponding to adult mice and newborn humans. Highly accurate reconstruction is achieved even when using a small fraction of the genomic MS loci (e.g., a tree of depth up to 400 cell divisions can be reconstructed with more than 90% accuracy using 10% of the genomic MS loci; see Figure 2). In MMR-deficient organisms, a few hundred MSs are sufficient for accurate reconstruction of complex cell lineage trees (Figure 2). MS loci that have excessive mutation rates should be avoided as they increase the likelihood of coincident and successive mutations. Simulations were performed for samples with up to 100 cells, because of the computational requirements of the phylogenetic analysis algorithm, but the results suggest that reconstruction scores may not decrease as the size of the cell sample increases.

### Silent Cell Divisions in a Newborn Human

In order to assess the extent of silent cell divisions, which might act as a limiting factor for reconstructing human cell lineage trees, we calculated the probability for a silent cell division and estimated the total number of cells in the tree for a newborn human. We found that in a single cell division the probability of a daughter cell acquiring no new mutations is less than 10^−21^. For estimating the total number of cells in the tree we created a model of human embryonic development that overestimates the number of cells and cell divisions, and thus can serve as a theoretical upper bound on the size of the cumulative cell lineage tree of a newborn human. We found that in the model, in more than 99.9% of newborns, there is at least one new mutation in each daughter cell in each cell division. This suggests that during human prenatal development, even a single silent cell division is unlikely to occur. As mentioned above, coincident mutations may cause erroneous tree reconstruction, but because there are no data on the topology and depth of newborn cumulative cell lineage trees, it is difficult to estimate their effect in this model.

### Experiments in Plants

**Figure 3:**
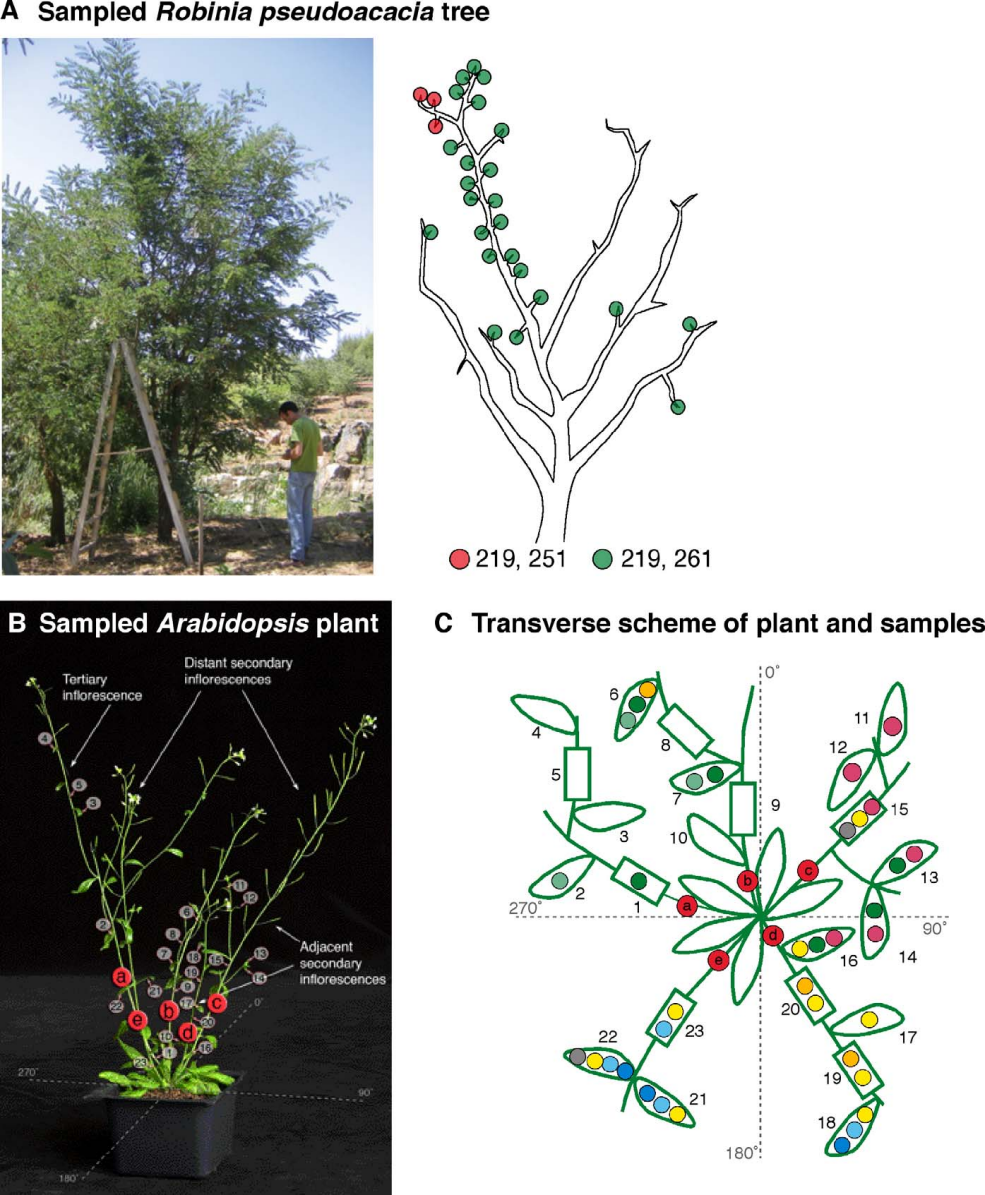
Analysis of Whole Organisms (A) Photograph and scheme of the *R. pseudoacacia* tree used for the lineage experiment. All three identically mutated samples (red) come from the same small branch. (B) *A. thaliana* plant used for the experiment. The location of each sample is indicated. (C) Transverse scheme of the *A. thaliana* plant showing all sampled stem (rectangles) and cauline leaf (ovals) tissues. Mutations that occurred in two or more samples are depicted by colored circles.

*C. elegans,* with its known cell lineage tree, may have provided an excellent in vivo control for our cell lineage inference procedure, except that its genome does not contain a sufficient number of MSs to allow precise reconstruction. Plants are a good model system for lineage studies at the tissue level because of their nearly invariant pattern of cell division and lack of cell migration ([39], p. 299), which results in a correlation between their physical and lineage distances. Our first evidence supporting this correlation was obtained from analysis of MS variability in wild-type *Robinia pseudoacacia* trees, which have been shown to have somatic mutations in an MS locus [40]. (Analysis of multiple MS loci in *R. pseudoacacia* is not currently possible since its genome has not been sequenced.) We located a tree with somatic MS variability and extracted DNA from 28 tissue samples. We found that 25 samples contained the same genotype, which was considered normal, and three samples contained a mutant genotype. These three samples were physically clustered on the same small branch, which contained only mutant samples (Figure 3A). This demonstrates that spontaneous somatic MS mutations in wild-type plants can be used as clonal markers. However, in some plants, such as *Pinus strobes,* the somatic mutations in MSs are rare [41]. In order to obtain sufficient mutations we therefore used MMR-deficient *Arabidopsis thaliana* for further experimental analysis.

Past work on *A. thaliana* analyzed genetic mutations that result in albino sectors [42,43], showing that tissues from the same organ are more likely to be clonally related than tissues from different organs [42], and that a small radial angle in the transverse plane between two samples increases the probability that they are clonally related [43].

In order to analyze genomic variability within a plant, we grew an MMR-deficient *A. thaliana* AtMSH2::TDNA mutant SALK_002708 [44], extracted DNA from 23 different tissue samples (Figure 3B), and amplified 22 MS loci for each DNA sample. We found that samples that shared a similar mutation in a particular MS locus tended to be physically adjacent (Figure 3C). This correlation between genetic and physical distance between the samples was statistically significant. Analysis of two other *A. thaliana* plants found far fewer somatic mutations and a weak correlation between genetic and radial distances (See Text S2 for details).

### An Automated Procedure for Lineage Reconstruction from DNA Samples

**Figure 4:**
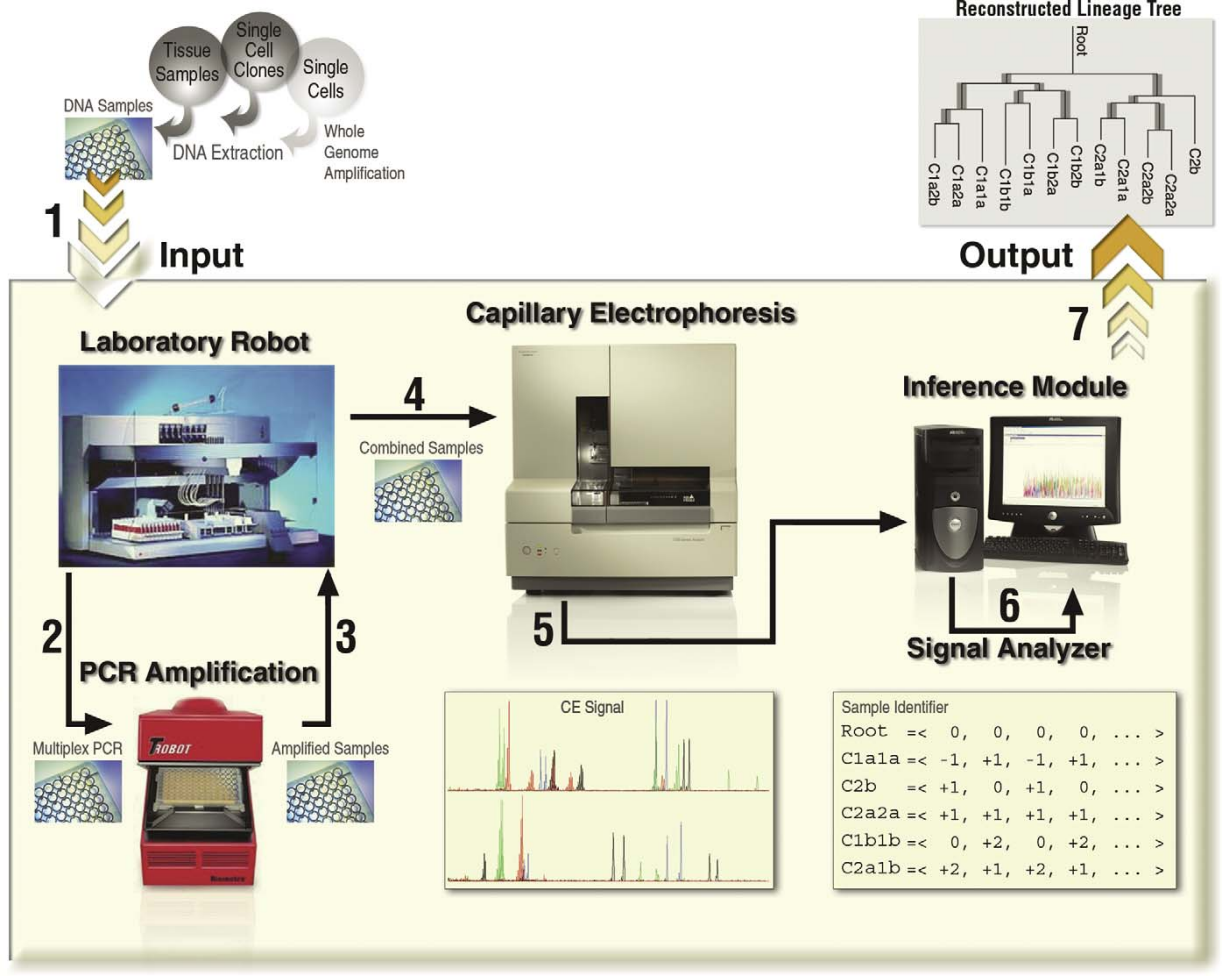
Automated Procedure for Lineage Tree Reconstruction The procedure accepts biological samples and PCR primers as input, and outputs a reconstructed lineage tree. It consists of a series of seven consecutive steps (numbered), during which the physical biological samples are “transformed” into digital data, which are then analyzed algorithmically. We built a hybrid in vitro/in silico automated system that performs steps 2–7 of the procedure (outlined), and used it to process DNA from tissue samples and single-cell clones. Incorporation of whole genome amplification techniques in the future may enable processing of single cells as well. For a detailed specification of the procedure, see Protocol S1.

We developed a procedure that takes as input a set of DNA samples, primers for MS loci, information on expected MS sizes, and information on PCR and capillary electrophoresis multiplexing compatibility between MS loci, and outputs a reconstructed cell lineage tree (with edge lengths) correlated with the DNA samples (Figure 4). The procedure involves common lab protocols, including PCR and capillary electrophoresis, and known algorithms, including a phylogenetic analysis algorithm. The resulting tree provides, in addition to the inferred topology, also depth information, representing the inferred number of cell divisions that occurred along each edge in the lineage tree, and confidence information. The procedure is oblivious to the DNA source and quality, which may be from clones of a single cell, from tissue samples, or from single cells. In this work we used DNA samples extracted from cell clones (with automatic signal analysis) and from heterogeneous tissue samples (with manual signal analysis). In principle, the procedure can work with DNA amplified from single cells [45], if provided in sufficient quantity. However, amplifying DNA from a single cell, reliably and in a sufficient quantity, is a technical challenge that has yet to be overcome. Therefore, at the moment, we focus on potential applications that can utilize cell clones. The DNA of cell clones represents the DNA of the founder cells sufficiently reliably to allow precise reconstruction of the lineage tree among those founder cells (see Materials and Methods and Text S3). However, not all cell types can be grown to clones of sufficient size for our current method to be applied without further DNA amplification.

We accomplished the procedure in a hybrid in vitro/in silico automated system, which operates as follows. A predetermined set of *n* MS loci is amplified from each sample by multiplex PCR and run on a capillary machine, yielding several histograms for each sample. A programmable laboratory robot augmented with a PCR machine performs the liquid handling for PCR, the PCR itself, and the preparation of the samples for the capillary machine. Sample analysis by the capillary machine produces histograms, which typically show two main peaks representing the allelic value of each MS, as well as a stutter pattern that is typical of PCR of MSs [38]. A computer program, developed by us, that utilizes a signal processing algorithm (see Protocol S1) resolves the stutter pattern and assigns relative allelic values to all the MS loci in all samples. To aid analysis, we select MS loci that are expected to produce little stutter upon PCR amplification. Subsequently, each sample is assigned an identifier—a vector of 2*n* elements corresponding to the 2*n* analyzed alleles. Each element (called relative allelic value) is a whole number equal to the difference between the number of repeats of that allele and the number of repeat units of the corresponding allele in an arbitrary reference sample (allelic crossover may occur; see Text S4). Finally, a computer program applies a phylogenetic algorithm to the set of sample identifiers and produces a reconstructed tree associated with the DNA samples.

### Cultured Cell Trees

**Figure 5:**
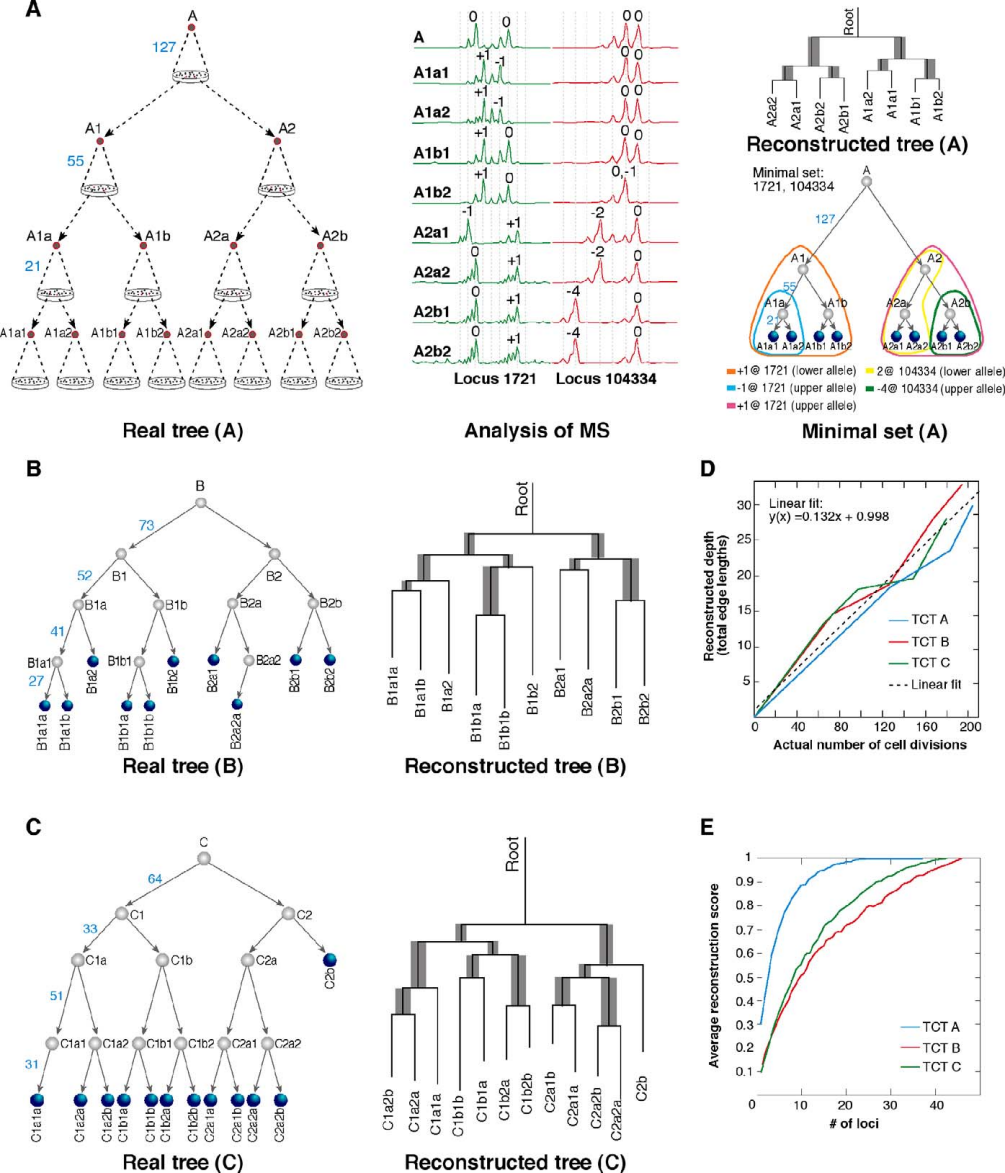
CCT Model System (A–C) A cell sample lineage tree with a predesigned topology is created by performing single-cell bottlenecks on all the nodes of the tree. Lineage analysis is performed on clones of the root and leaf cells. Three CCTs (A–C) were created using LS174T cells that display MS instability. All topologies were reconstructed precisely. Edge lengths are drawn in proportion to the output of the algorithm. Gray edges represent correct partitions according to the Penny and Hendy tree comparison algorithm [29], and their width represents the bootstrap value [29] (*n =* 1,000) of the edge. A minimal set of loci yielding perfect reconstruction was found for each CCT (each colored contour represents a different mutation shared by the encircled nodes; see also Figure S2). (D) There is a linear correlation (*R* ^2^ = 0.955) between reconstructed and actual node depths. (E) Reconstruction scores of CCTs A–C using random subsets of MS loci of increasing sizes (average of 500).

To quantitatively evaluate the cell lineage tree reconstruction procedure, we cultured ex vivo cell trees with known topologies and well-estimated edge lengths, called cultured cell trees (CCTs). We constructed three CCTs (A–C; Figure 5A–5C) using human adenocarcinoma cells (culture LS174T, European Collection of Cell Cultures), which have a mutation in a key MMR gene [46] and high MS mutation rates [47]. We chose a set of 51 MS loci of various repeat types and various numbers of repeats (see Table S1 and Text S3B for selection criteria). DNA samples obtained from the root and leaf nodes were fed into the cell lineage tree reconstruction procedure, yielding a reconstructed tree for each CCT (see Table S2 for all cell identifiers). Reconstructions were performed using the neighbour-joining (NJ) [29] phylogenetic algorithm. In all cases the topology of the CCT was reconstructed precisely; thus, the correct topology was found out of a total of (A) 135,135, (B) 34,459,425, and (C) 13,749,310,575 possible topologies for the 8, 10, and 12 leaves of CCTs A, B, and C, respectively (Figure 5A–5C). The edge lengths in the reconstructed trees were in linear correlation to the actual number of cell divisions in the CCTs (Figure 5D; *R* ^2^ = 0.955). Furthermore, reconstructions of the CCTs without using root identifiers (Figure S1) yielded perfect scores for CCTs A and C, and a score of 7/8 for CCT B (the incorrect edge is colored red in Figure S1), suggesting that accurate reconstruction is feasible from the extant cells alone. Finally, for each CCT we found a minimal set of loci that yielded correct reconstruction using NJ (Figure 5A; colored contours in Figure S2), and analyzed how many loci were needed, on average, for precise reconstruction. We found that CCT A, being simpler than trees B and C, indeed requires fewer loci (Figure 5E). These results suggest that in MMR-deficient organisms, complex lineage trees may be reconstructed using a small set of MS loci. CCTs serve as a controlled system for phylogenetic analysis, and also may provide exact numerical data regarding the rates, nature, and correlation of mutations, allowing the assessment of the validity of MS mutation models.

## Discussion

We discovered that somatic mutations in higher organisms carry enough information to enable precise reconstruction of the entire organism cell lineage tree. We demonstrated the practical utility of the discovery by developing a prototype automated procedure for the reconstruction of cell lineage trees from DNA samples.

In the short term, small-scale projects utilizing this discovery and its associated procedure may aim to gain preliminary understanding of partial lineage trees associated with different organs or systems, by analyzing cell samples containing only dozens or hundreds of cells. In addition, analysis of the development of cancer using this method may provide immediate benefits. Cancer analysis may not require the perfection of single-cell methods, since clonal tissue samples may be obtainable from solid tumors.

In the longer term, with the improvement of DNA sequencing technologies [48], these results may inspire the initiation of a “Human Cell Lineage Project,” whose aim would be to reconstruct an entire human cell lineage tree. A precursor project, which may face fewer hurdles, would be a “Mouse Cell Lineage Project.” Both projects would require multidisciplinary teams, with members familiar with different organs or biological subsystems, but either project would benefit from the teams working on the same individual organism, since accumulated mutation information regarding the same individual could greatly improve the precision of the overall tree reconstruction process. Still, as in the Human Genome Project, diversity would be needed to separate incidental from essential properties of the organism cell lineage tree.

## Materials and Methods

### Number of MS loci and estimation of the number and rate of MS mutations.

We downloaded the human (build 35) and mouse (build 33) genomes from UCSC Genome Bioinformatics (http://hgdownload.cse.ucsc.edu/downloads.html). We wrote a MATLAB (MathWorks, Natick, Massachusetts, United States) program for searching MSs in any sequenced genome and used it to search for all mono- to hexanucleotide MSs in human and mouse that were nine uninterrupted repeats or longer. For any repeat unit (e.g., AAG) its frame shifts (AGA, GAA) were not searched, so results are a slight underestimate. See Tables S3 and S4 for data.

For estimation of the number of MS mutations in each cell division in human, we obtained from the literature [49] the approximated rates of mutations in human MSs per human generation, as a function of the length of the MS (number of uninterrupted tandem repeat units). Although the rate of mutations in MS loci is also dependent to a great extent on the specific locus examined [31], in our analysis we assumed as a first approximation that the average mutation rate is the mutation rate obtained from [49]. Mutation rates in [49] are given per human generation. These mutation rates were transformed to rates per cell division by dividing them by 186.5, which is the average of the approximated number of cell divisions in human male and female generations (350 and 23, respectively; see [49]). Because the mutation rates of MSs with 9–15 repeat units seem to increase exponentially with MS length, we used MATLAB to calculate a linear fit of the logarithm of these mutation rates. From this linear fit we obtained the mutation rates for MSs with 9–15 repeat units. The linear fit gives


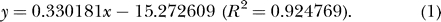


The mutation-rate function for MSs with 9–15 repeat units is therefore


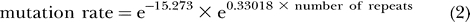


where e is the basis of the natural logarithm. For all MSs with less than nine repeat units, we made a conservative assumption that their rate of mutation is zero. Because of the lack of information regarding mutation rates in MSs with more than 15 repeat units, we made another conservative assumption that the mutation rates of all such loci are the same as for loci with 15 repeat units. Therefore, our estimated mutation rates for short and long MSs most likely represent an underestimate of the actual rate. We sorted the human MSs according to their length, and for each length we computed the expected number of mutations acquired by a daughter cell in a single cell division by multiplying the mutation rate by the number of MSs. The total number of expected MS mutations was computed by summing the expected number of mutations in each length category. See complete data in Table S3.

In contrast to the information regarding human MS mutation rates, there are fewer published data regarding mouse MS mutation rates, and data from different sources may be inconsistent. Comparison of data from several studies of human [50,51] and mouse [52,53] germ line mutations reveals that the rate of MS mutations per organism generation in mouse is 1–10 times lower than the human rate. In a model of MS mutations based on equilibrium distributions of MS repeat lengths [54], the rate of mouse MS mutations per organism generation is about five times higher than the corresponding human rate. Mice have faster life cycles than humans, and consequently the number of cell divisions per mouse generation is smaller than in humans. It is estimated that mice have approximately 6.5-fold fewer cell divisions per organism generation than humans [55]. Incorporating the data on MS mutations per organism generation and the estimated numbers of cell divisions, the rate of MS mutations per cell division in mice seems to be from about 1.5 times lower to about 30 times higher than the corresponding human rate. In our calculation of the expected number of mutations in mice, we make a conservative assumption that the rate of mutations is equal to the corresponding rate in humans (described above). Based on this assumption and the numbers of mouse MS alleles, we calculate the expected number of mutations in a similar fashion to the corresponding calculations in human MS (described above). See data in Table S4.

Our mathematical analysis, simulations, and the reconstruction of CCTs assume a uniform MS mutation rate across tissue types, as there is no sufficient knowledge at present to assign different somatic MS mutation rates to different tissue types.

### Proof of theorem 1.

The simplifying assumptions underlying the uniform model make the calculation of some key quantities (which we call “signal,” “noise,” and “loss,” as indication of their effect on our eventual reconstruction algorithm) rather straightforward. We provide such calculations now (omitting some details). For signal, the expected number of mutations per edge of the extant tree is *m* × *p* = 50. The probability that the number of mutations on an edge is *t* < 50 behaves roughly like 50*^t^* × e^−50^/*t*! (here e is the basis of the natural logarithm). For noise, we estimate the number of accidental coincident mutations as follows. Given two branches of length *b,* the expected number of coincident mutations along these branches is roughly (50*b*)^2^/2*m*. When *b* = 40 (which is the maximum that we shall consider in this manuscript), this number is one. The probability that there are *t* coincident mutations in two branches of length 40 behaves roughly like 1/e × 1/*t*! For loss, successive mutations at the same locus may result in a loss of the signal. We estimate the extent of this loss as follows. Essentially, the worst case is when two leaves share a path of length *d*/2 = 20 (on which they are expected to have 1,000 common mutations), and then continue separately up to depth 40. They will each have roughly 1,000 more mutations, so one of the common mutations is expected to be undone. On the other hand, they are expected to share 1/4 of a coincident mutation, which somewhat compensates for the lost mutation. It turns out that for our analysis (with *d* = 40) the effect of lost mutations is negligible, and we will ignore it altogether. (Ignoring lost signal is further justified by the fact that the worst case for lost signal appears in tree configurations that are very different from those that give the worst case for coincident mutations.)

We now describe the reconstruction algorithm that we analyzed. This is a new algorithm, which we call the “triplet algorithm,” designed to facilitate the proof. This algorithm is chosen here because its analysis is simple, but we do not necessarily advocate its use in practice. We suspect that similar (and perhaps even better) results are true for other algorithms as well.

The basic primitive of the triplet algorithm is a “triplet subroutine.” Given identifiers for three cells (say, A, B, and C), the triplet subroutine counts for every pair of cells the number of common mutations, namely, the number of loci in which the two cells have the same label, and moreover, this label is different from the corresponding label of the root. The pair of cells that maximize this count (say, A and B) are output by the triplet subroutine. We say that the triplet subroutine is “successful” if the pair of cells that it outputs is the one that has the longer common branch (or equivalently, the deeper common ancestor).

The triplet subroutine will be successful unless there is some value of *t* such that there were at most *t* mutations along the branch common only to A and B, and at least *t* accidental coincident mutations between B and C (or between A and C). The probability of this event is roughly 50*^t^* × e^−51^ × (1/*t*!)^2^, which is maximized when *t* = 7, giving roughly 2.2 × 10^−18^. Summing over all values of *t* (using the fact that there is an exponential drop as we move away from *t* = 7), the total probability of not being successful is around 10^−17^. Hence one can execute the triplet subroutine on 10^17^ arbitrary (not just random!) triplets, and still be likely to be successful in all executions.

We now describe the triplet algorithm. View every cell as a vertex in an auxiliary graph G. In an execution of a triplet subroutine that outputs cells A and B (say, on input A, B, and C), put an edge between A and B. As long as there are more than two connected components in the graph G, pick three vertices from three different components and execute a triplet subroutine on them, thereby adding an edge to the graph and decreasing the number of connected components by one. After *m −* 1 steps, two connected components remain. Each one of them necessarily corresponds to a subtree of depth at most *d −* 1. The condensed version of each of the subtrees can be inferred separately by repeating the above procedure. Hence, at most (and in most cases, less than) *d* × *m* successful executions of the triplet subroutine suffice.

We have just shown that for every extant tree of depth *d* and *m* extant cells, *d* × *m* consecutive successful executions of the triplet subroutine guarantee that the triplet algorithm outputs the true condensed tree. This suffices in order to prove our theorem, because 10^17^ is much larger than 40 × 2^40^. In fact, the ratio between these numbers is such that the probability that the tree is constructed with no error is greater than 0.9995. This completes our proof.

### Computer simulations.

The simulations demonstrate, first, that human wild-type MS mutations enable accurate reconstruction of cell lineage trees and, second, that with higher mutation rates, as in MMR-deficient cells, cell lineage trees can be accurately reconstructed with no more than 800 MS loci (a kit containing 800 primer pairs for human MS amplification is commercially available).

The simulation proceeds as follows. A random tree is generated according to chosen topology type, maximal depth, and number of leaves. MS mutations are simulated according to number of loci and mutation rates, and leaf identifiers are generated. A lineage inference algorithm reconstructs the lineage tree. The inferred tree is compared with the generated tree, and the result is scored. Mutations and tree inference are performed ten times for each generated tree. For each depth, five random trees from each topology type are generated.

Since we do not usually have prior knowledge of the real organism's tree topology and branch lengths, we simulated two types of random trees that reflect topology space variability to a reasonable extent (see Figure 2). For the simulation, we generated trees with 32 leaves and various depth limits that reflect limits on the number of cell divisions from the root (i.e., the zygote). Each tree had 32 leaves, a large enough number to reflect the tree topology. Increasing the number of leaves does not necessarily increase inference difficulty since it adds information. In our simulations, increasing the number of leaves from 32 to 100 did not affect the average score.

Type I random trees have a random binary path, and are generated as follows. Generate LEAF _ NUMBER of unique nonoverlapping binary strings, each of a random length of up to MAX _ DEPTH bits. Each string represents a path in a binary tree leading to a leaf. In such a tree the least common ancestor of any pair of leaves is usually relatively close to the root. There is only a (1/2)*^n^* chance that two random binary strings of length *n* will be the same; therefore, within a tree of 32 samples and depth 100, most leaves will split within the first six tree levels. Such a tree is difficult to infer since the mutation signal on the tree internal edges is low and the mutation noise (i.e., coincident mutations on long paths) is high.

Trees of type II are generated by random node addition, as follows. The tree is initialized with two paths of random length up to MAX _ DEPTH that start at the root and lead to two leaves. An iteration adds a leaf by randomly picking a path, and on it randomly picking an internal node as the source of the new path. The depth of the new leaf is determined randomly between (new internal node + 1) and MAX _  DEPTH. Leaves are added until LEAF _ NUMBER is obtained. The procedure generates a variety of tree topologies with branches at various depths. The procedure often produces nonbalanced trees. In this family of trees, increasing the maximum depth does not always result in an increase of noise over signal since internal branches are often deep.

Mutations in MS loci were simulated by a stepwise model. The root was assigned a vector of zeros of the size of the simulated ALLELE _ NUMBER. An ALLELE _ MUTATION _ RATE, which is the probability of each MS locus mutating in a single cell division, was chosen. Then in each simulated cell division, each locus could mutate by increasing or decreasing the repeat number according to its assigned probability. Starting with the root identifier we generated the identifier of all tree nodes and leaves by simulating mutations as described above.

In simulated MMR-deficient cells we used mutation rates not higher than one per 100 cell divisions, according to Table 1. A future algorithm might use loci of various mutation rates, for example, using slow and reliable MS loci to obtain the coarse topology and then using the faster loci to infer local subtrees for which slow loci do not provide enough information.

**Table 1:**
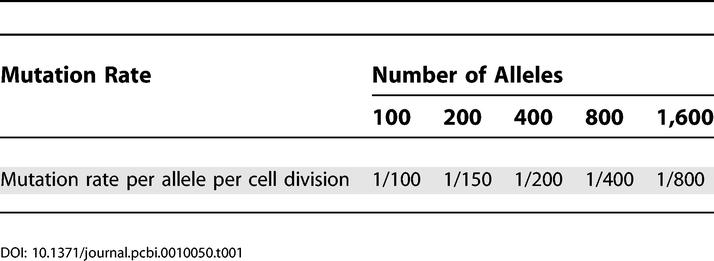
Mutation Rates in MMR-Deficient Human Simulations

Lineage tree inference was done with the NJ algorithm [29], which uses a distance matrix as input. We used the “equal or not” distance function, which increases the distance between two identifiers by one for each locus that differs. Inference using a maximum parsimony algorithm was also tried, with similar results.

The generated tree and the reconstructed tree were compared using Penny and Hendy's topological distance algorithm [29] (implemented using MATLAB). In this algorithm, the removal of each internal edge partitions the root and leaves into two groups. We assigned a score equal to the ratio of partitions, which was equal in the two trees to the total number of partitions. This scoring is rather strict as it might drop considerably with even single leaf misplacement.

Simulations were performed for samples with up to 100 cells, because of the computational resources required by the phylogenetic analysis algorithm.

### Silent cell divisions in humans.

The probability for no mutations in each daughter cell in each length category was calculated by the formula


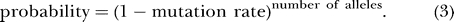


The probability for no mutations in all loci was calculated by multiplying the probabilities for no mutations in all length categories (see data in Table S5).

In order to estimate the total number of cells in a human neonatal cell lineage tree, we developed a model of human wild-type development**.** This is an overestimate model, which is intended to contain a larger depth and a larger number of cell divisions than the real (unknown) tree. In this model, development starts from a single cell, the zygote, in a series of 46 binary cell divisions, producing a binary tree with a full depth of 46, which has approximately 10^14^ leaves and approximately 10^14^ internal nodes, and hence has approximately 2 × 10^14^ nodes altogether (according to published data the adult human has about 10^14^ cells). This series of 46 divisions lasts for 23 d because each cell cycle is exactly 12 h long (according to published data the cell cycle in early human embryogenesis is 12–24 h). From this point on, there are additional 486 cycles of 12 h in 243 d until birth. In each cycle, each cell divides with a probability of 0.5 and dies with a probability of 0.5. Therefore, the number of living cells remains relatively constant from day 23 to day 266 (birth) at about 10^14^, and in each day 2 × 10^14^ cells are produced. The total number of cells produced during this process, and therefore the total number of nodes in the complete cell lineage tree at birth, is approximately 2 × 10^14^ + 486 × 10^14^ = 4.9 × 10^16^.

The probability for at least one new MS mutation in every cell in the human neonatal cell lineage tree was calculated by the formula (probability for no MS mutations in a single daughter cell)^total number of cells^. This calculation gives


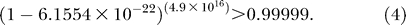


### Experiments in plants.

DNA from *A. thaliana* and *R. pseudoacacia* was extracted using the Extract-N-Amp kit (Sigma, St. Louis, Missouri, United States), and amplified according to the kit instructions. A list of primers for *R. pseudoacacia* and *A. thaliana* is given in Tables S6 and S7, respectively. Amplified products were run on a capillary electrophoresis machine (ABI Prism, Avant-3100, Applied Biosystems, Foster City, California, United States). Mutations were determined by manual comparison of capillary histograms.

Individuals of *A. thaliana* AtMSH2::TDNA mutant SALK_002708 (seeds kindly provided by J. Leonard) were grown in a growth room under long-day conditions. All *A. thaliana* plants were verified as mutant as described in [44].

### CCTs.

Primers for most MS loci were designed using Primer3 (http://frodo.wi.mit.edu/cgi-bin/primer3/primer3_www.cgi) with the following parameters changed from default: Primer size = 20,22,27 (minimum, optimal, maximum); Primer Tm = 62 °C, 65 °C, 68 °C; Max Tm difference = 2.5 °C; CG clamp = 1. Some primers were taken from STRbase (http://www.cstl.nist.gov/biotech/strbase/).

CCTs were created using LS174T human colon adenocarcinoma cells, which were obtained from the European Collection of Cell Cultures (Salisbury, United Kingdom) and were grown in medium containing EMEM (Eagle's minimum essential medium, in Earle's balanced salt solution, GIBCO, San Diego, California, United States), 2 mM glutamine, 1% nonessential amino acids, 10% fetal bovine serum, and 1% penicillin-streptomycin. We estimated that LS174T cells divide every 1.5 d according to the frequency of routine plate passages. We created CCTs as follows. Initially, a single cell was isolated from a cell stock and was defined as the tree root. This cell was allowed to proliferate for a desired number of cell divisions (passages were performed when required). Then, two cells were isolated from the root progeny, and were defined as its daughter cells in the tree. This procedure was continued for each daughter cell, creating the granddaughter cells, etc., until the entire tree was grown. The tree root and leaf cells were cloned in plates, and lineage analysis was performed on DNA obtained from these clones. Lineage analysis performed on clones is expected to yield the same results as analysis on the founder cells of the clones (see Text S3A). Clones from single cells were created as follows: (1) trypsinizing and lifting cells from semiconfluent plates, (2) thrusting the cells ten times through a 1-μm mesh (Sefar, Heiden, Switzerland), (3) verifying by microscope that 99% or more of the cells were not attached to other cells, (4) diluting the cells with ratios ranging between 1:5,000 and 1:100,000 and spreading the cells on new plates, (5) waiting for single cells to form small islands (about 2–3 wk), and (6) lifting islands to new plates using cloning cylinders (Sigma). DNA was extracted from clones of all cells corresponding to nodes of the CCTs using Wizard SV Genomic Purification System (Promega, Fitchburg, Wisconsin, United States). Cells from all nodes of the CCTs were frozen in liquid nitrogen using a freezing medium containing 90% fetal bovine serum and 10% DMSO. Lineage reconstruction from CCT DNA samples (root and leaves only) was performed according to the automated procedure, as described in Protocol S1.

Figure S2 shows reconstructed trees for CCTs A–C without using the root for reconstruction. All reconstructions were performed using NJ (with the “equal or not” distance function). The unrooted trees outputted by NJ were rooted at the midpoint of the longest path from among all possible pathways between any two leaf nodes. Reconstructions of CCTs A and C were perfect, and a score of 7/8 was achieved for CCT B (Figure S1). The identifier of the zygote or root of a tree may be deduced in one of the following manners: (1) deduction from parental identifiers or (2) deduction from the most common allele. Deduction from parental identifiers (deduction of zygote identifier) is performed as follows. When performing lineage analysis on tissues from MMR-deficient organisms, the organism should be produced by a cross between two animals heterozygous for a mutation in an MMR gene (e.g., *Mlh1* ^+/−^), with each parent from a different inbred line. Animals that are heterozygous for a mutant MMR gene have normal or slightly elevated MS mutation rates. A cross between two such animals produces (with a frequency of 1:4) an animal that is homozygous for the mutant gene, with greatly elevated MS mutation rates. In order to deduce the identifier of the root (zygote) of such an organism, which is used in an experiment, the identifiers of its parents should be obtained. Because the parents come from inbred lines, they are homozygous at each MS locus and therefore deducing the identifier of the zygote is straightforward. The deduced identifier is very close (and when analyzing a few hundred MS loci may be identical) to the actual identifier because somatic MS mutations in the parents are very rare. It is important to note that this procedure deduces the identifier of the zygote of the organism, which may or may not be identical to the root of the reconstructed tree.

Deduction from the most common allele (deduction of root identifier) is performed as follows. In this procedure, the most common allele is determined for each MS locus in the population of sampled cells, and this value is assigned to the root identifier. Thus, the root identifier consists of the most common values in the cell population. In balanced trees that are not too deep, the deduced identifier will be very close (and may be identical) to the actual root identifier. However, in unbalanced (nonsymmetric) trees, this procedure will result in the deduced identifier being “tilted” towards the larger branch, and in deep trees the deduced identifier may differ from the actual identifier in MS loci that accumulate mutations in a nonsymmetric fashion. For example, in an MS locus that is biased towards MS contraction, the deduced identifier value may be smaller than the actual value. It is important to note that this procedure deduces the identifier of the root of the tree, which is not necessarily the zygote of the organism.

## Supporting Information

Figure S1 Reconstructed Trees for CCTs A–C without Using the Root for Reconstruction (56 KB JPG)

Figure S2 Example Minimal Sets of Loci Yielding Perfect Reconstruction of CCTs B and C (93 KB JPG)

Protocol S1 Specification for the Automated Procedure for Lineage Reconstruction from DNA Samples A complete description of the protocol for reconstructing lineage trees from DNA samples, including the capillary histogram signal analysis algorithm and tree reconstruction and scoring algorithms.
(474 KB DOC)

Table S1 List of MS Loci Used for the CCT Model System (89 KB DOC)

Table S2 Cell Identifiers for CCTs A–C (88 KB DOC)

Table S3 Estimated Number of MS Mutations in Each Cell Division for Human (27 KB DOC)

Table S4 Estimated Number of MS Mutations in Each Cell Division for Mouse (26 KB DOC)

Table S5 Silent Cell Divisions in Human (27 KB DOC)

Table S6 List of MS Loci Used for *R. pseudoacacia*
(28 KB DOC)

Table S7 List of MS Loci Used for *A. thaliana*
(50 KB DOC)

Text S1 Lineage Analysis at the Single Cell and Tissue Levels (48 KB DOC)

Text S2 Full *A. thaliana* Results (165 KB DOC)

Text S3 Reconstruction Using Cell Clones and MS Selection Criteria (A) Reconstructing cell lineage trees from DNA extracted from cell clones.
(B) Selection criteria for MSs.
(29 KB DOC)

Text S4 Ignoring the Effect of Allelic Crossovers (27 KB DOC)

## Abbreviations

CCT: cultured cell tree
MMR: mismatch repair
MS: microsatellite
NJ: neighbor joining
