# Supplementary figures and images for: Genomic Variability within an Organism Exposes Its Cell Lineage Tree
Source: PLoS Comput Biol. 2005 Oct 28;1(5):e50. doi: 10.1371/journal.pcbi.0010050 (PMC1274291; doi:10.1371/journal.pcbi.0010050)

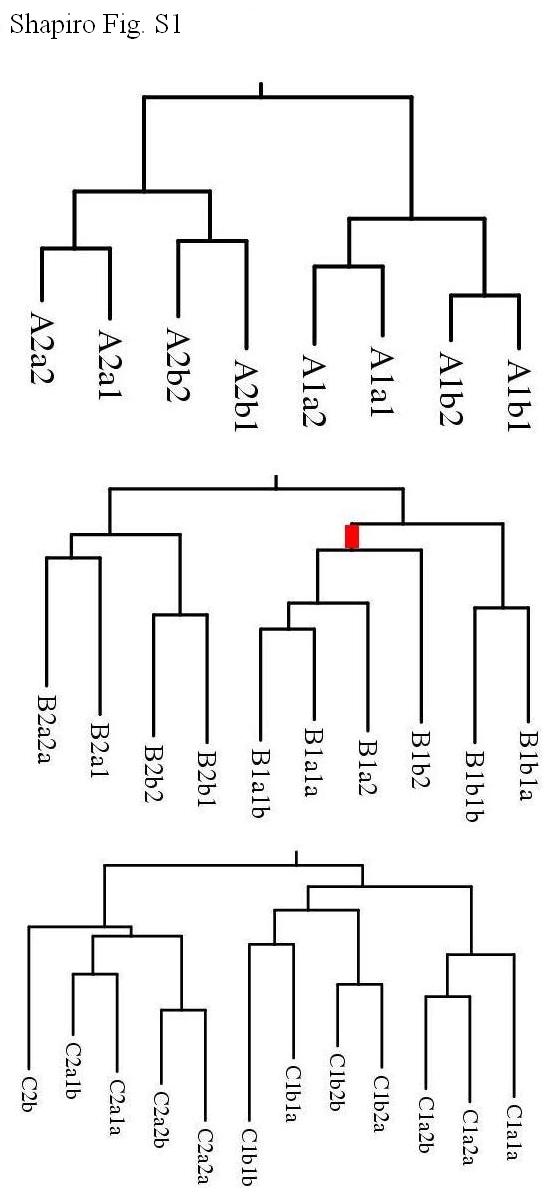

Supplement: Figure S1 — (56 KB JPG) [file pcbi.0010050.sg001.jpg]

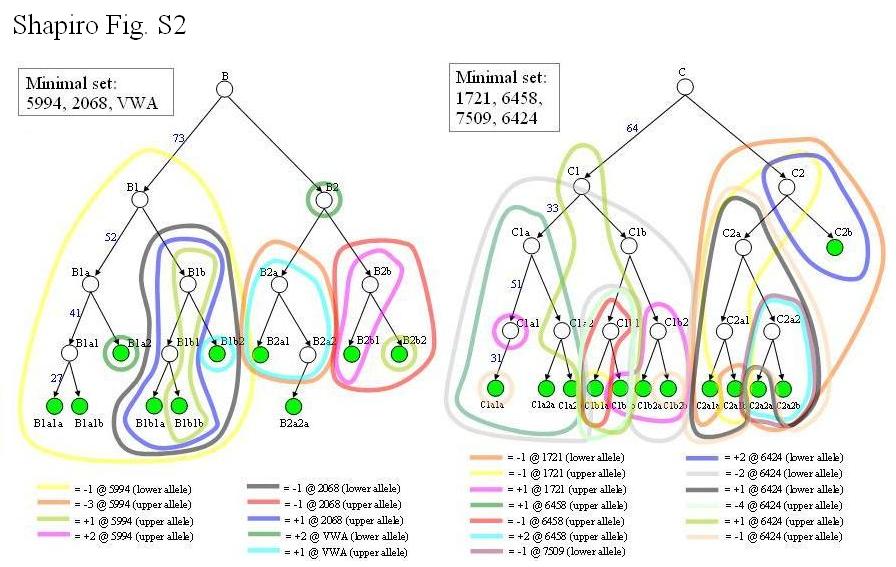

Supplement: Figure S2 — (93 KB JPG) [file pcbi.0010050.sg002.jpg]
