# Supplementary material for: Genomic Variability within an Organism Exposes Its Cell Lineage Tree
Source: PLoS Comput Biol. 2005 Oct 28;1(5):e50. doi: 10.1371/journal.pcbi.0010050 (PMC1274291; doi:10.1371/journal.pcbi.0010050)
Supplement: Protocol S1 — A complete description of the protocol for reconstructing lineage trees from DNA samples, including the capillary histogram signal analysis algorithm and tree reconstruction and scoring algorithms. (474 KB DOC) [file pcbi.0010050.sd001.doc]

### Protocol S1. Specification for the automated procedure for lineage reconstruction from DNA samples


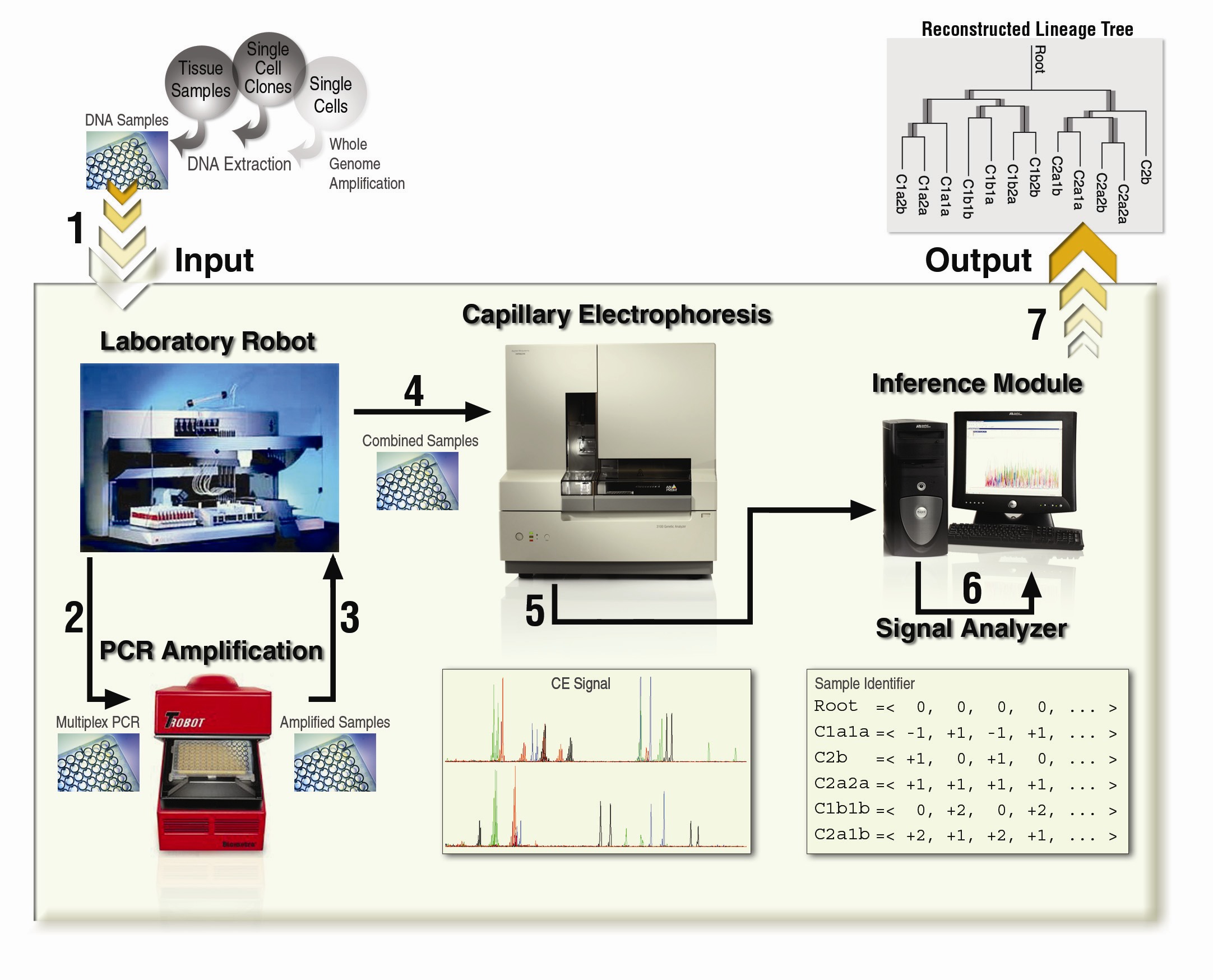


**Components Specifications**

1. **Robotic PCR preparation**

Input:

- Plate with extracted DNA samples (Figure, Channel 1)
- Plate with MS loci primers set
- Tube of PCR reaction mix (dNTPs, hot-start enzyme, buffer)
- Trough of DDW
- File describing the multiplex PCR primer groups
  - List of primer pairs
  - Groups and concentrations of multiplex PCR
- File of the DNA sample name

Output:

- A PCR plate with amplification reaction of a set of samples on a set of MS loci (Figure, Channel 2)

Description:

The plates and tubes are loaded to the robot work table. A program compiles the input files and generates a robot program which is executed by the Tecan genesis robot.

1. **PCR amplification**

Input:

- PCR plate with amplification reaction of a set of samples on a set of MS loci. (Figure, Channel 2)
- PCR program

Output:

- PCR plate with amplified MS loci for each sample. (Figure, Channel 3)

Description:

The PCR plate is loaded by the robot to robotic thermo-cycler (Biometra TRobot). The PCR program is then called and executed automatically.

Amplification: In each amplification reaction 3-6 MS loci were amplified together in a multiplex reaction. CCT reactions were performed in 50µl including 100-125ng DNA, 0.1-0.5µM of each primer, 0.2mM of each dNTP, and 1.25U of AmpliTaq Gold polymerase (Applied Biosystems). Thermal cycling conditions for CCT were: (i) 95°C for 10 min, (ii) 35 cycles: 95°C for 1 min, 58°C for 1 min, 72°C for 1 min, (iii) 72°C for 45 min, (iv) 16°C hold.

1. **Robotic Capillary Electrophoresis sample preparation**

Input:

- PCR plate with amplified MS loci for each sample (Figure, Channel 3)
- File describing the multiplex PCR primer groups
  - Dilution rate of groups
  - Grouping for CE runs
- File of the DNA sample name
- Tube with formamide + Liz standard size marker (Applied Biosystems)
- Trough of DDW
- ABI 3100-Avant plate

Output:

- CE plate with samples ready for Capillary Electrophoresis (Figure, Channel 4)
- CE plate file describing the amplified samples and loci groups in each well (Figure, Channel 4)

Description:

The amplified sample plate is off loaded by the robot from the thermo-cycler and placed on the worktable. Each reaction is diluted and combined with other amplification reaction of the same sample on other loci. Dilution rates and combined groups are taken from the loci data file. 3µl of the combined diluted sample is than diluted with 15µl of formamide Liz marker in the CE plate. A program then generates a CE plate file.

1. **Capillary Electrophoresis machine**

Input:

- CE plate with samples ready for Capillary Electrophoresis (Figure, Channel 4)
- CE plate file describing the amplified samples and loci groups in each well (Figure, Channel 4)

Output:

- File with CE signal sizing table as exported from GeneMapper (Figure, Channel 5)

Description:

Amplified products were run on a capillary electrophoresis machine (ABI Prism 3100-Avant Genetic Analyzer, Applied Biosystems) and fragment analysis was performed using the GeneMapper v3.5 software accompanying the machine. This package enables high resolution analysis and can separate between amplified products with a 1bp difference for fragments lengths up to several hundred of bps. The GeneMapper sizing table was exported to a file.

1. **Capillary Electrophoresis signal analyzer**

Input:

- File with CE signal sizing table as exported from GeneMapper (Figure, Channel 5)
- Loci database including the following:
  - Loci names
  - Loci MS repeat lengths
  - Reference length for both alleles (obtained manually from arbitrary sample, usually the root)
  - Loci CE colors

Output:

- Sample identifier data file. (Figure, Channel 6)
  - Each sample is assigned a identifier, which is a vector of the relative allelic values for all loci on both alleles
  - Null value is assigned if amplification failed for a specific allele.
  - Reports of signal analysis problems for manual control.

Description:

The signal analyzer is software that converts the CE signal to a mathematical identifier for each sample. Due to a large amount of capillary electrophoresis data and since MS amplification often results a complex stutter pattern of several peaks there is a need for automatic CE signal processing algorithm. We have developed an automatic algorithm that detects what is the relative allelic value of each allele of each sample and resolves the stutter pattern and signal overlaps between two close alleles. The algorithm was calibrated according to a manual analysis of CCT A and we have reached 97% accuracy when compared to the manual analysis. CCT B was analyzed completely by the automatic software. CCT C was analyzed by the automatic algorithm software and results where compared to a complete manual analysis with accuracy of 91%. The algorithm generates relative allelic values for each sample and loci assuming the upper signal refers to the upper allele and the lower signal refers to the lower allele and ignoring the possibility of allelic crossover which is assumed to be rare (as discussed in the mathematical proofs section).

Algorithm Description:

- Signal-Retrieval: Retrieve the correct capillary electrophoresis histogram (CEH) of both alleles of the locus according to the CE-run file and the locus color and typical reference length for each allele. All relevant peaks within a given window of WINDOW_SIZE repeats around the typical length of the allele are retrieved. If no signal exists a null value is assigned and a warning is given.
- Binning: The CEH is then binned according to the size of the repeat length. In each bin the maximal peak value is selected as the bin value. Each bin is associated with a allelic value (e.g., -2, +1, 0) relative to the reference.
- Overlapping signal: The method detects whether there is an overlap between the CEH of upper allele and lower allele. If no overlap exists the highest peak of each allele is selected and its bin relative allelic value is assigned to the identifier.
- Candidate Peak Detection: The peaks that are less than PEAK_THRESHOLD of the maximal peak within the window are considered noise and are filtered. hen the method identifies candidate peaks that are local maxima within the signal window. If more than two candidates exist the two candidates that are closer to the reference are selected.
- Assigning Candidates Peaks to Alleles:
  - If any of the alleles have two candidates the upper candidate allelic value is assigned to the upper allele and the lower candidate allelic value is assigned to the lower allele. In this case if the other allele has only one candidate peak its relative allelic value is assigned for this allele.
  - If one candidate exist for both alleles the following are possible:
    - The peaks are different from each other. In this case the peak is selected and its relative allelic value is assigned.
    - The peaks are the same, a case which requires stutter pattern resolution as follows: the two following conditions are evaluated:
      - Condition 1: The peak adjacent to the max from the right is bigger than UPPER_THRESHOLD of the max peak.
      - Condition 2: The peak adjacent to the max from the left is bigger than LOWER_THRESHOLD of the max peak.
      - If both conditions are false a homozygote sample is assumed and relative allelic values are assigned accordingly.
      - If condition 1 is true the max peak is selected for the lower allele and the peak adjacent from the right is selected for the upper allele. If also condition 2 is true than a warning is issued and the case is considered ambiguous.
      - If condition 1 is false and condition 2 is true the max peak is selected for the upper allele and the peak adjacent from the left is selected for the lower allele.
- Single allele Flag: There are cases where only one allele is amplified and creates the impression of homozygote sample. It is highly unlikely that mutation of homozygote will change simultaneously in both alleles to the same direction in all samples. In such a case the signal is considered as single allele and zero is assigned as the other relative allelic value for all samples. This is done to avoid overestimation of mutations that occur in one allele and consider them twice for inference purposes.

Parameters Defaults:

- PEAK_THRESHOLD 0.25
- UPPER_THRESHOLD 0.3
- LOWER_THRESHOLD 0.75
- WINDOW_SIZE 4

1. **Inference Module**

Input:

- Sample identifier data file. (Figure Channel 6)

Output:

- A lineage relation tree of the input DNA samples including bootstrap values of internal tree edges.

Description:

**Tree reconstruction:** for each tree (CCT or simulated tree) a distance matrix was generated by applying a distance function to all pairs of identifiers from the set of root and all leaves. We used the ‘equal or not’ distance function, which is the sum of differing values between two identifiers (we are aware that a distance function taking into account the step-wise nature of MS mutations may yield better results, but decided at this stage to use a function that assumes as little as possible). This matrix was used as input to a program implementation the phylogenetic algorithm Neighbor-Joining (NJ) (Our MATLAB implementation as well as a web-based implementation: <http://bioweb.pasteur.fr/seqanal/phylogeny/phylip-uk.html>, were used). The un-rooted output tree was rooted from the node corresponding to the root, yielding the reconstructed tree (which also includes edge lengths). Tree reconstructions without using the root were performed in a similar fashion, yet instead of rooting the tree from the root, the tree was rooted at the midpoint of the longest path from among all possible pathways between any two leaf nodes. **Tree comparison:** in each reconstruction (CCT or simulated tree) the reconstructed tree and real tree were compared using Penny and Hendy’s topological distance algorithm (implemented using MATLAB). In this algorithm each internal edge confers a partitioning of the tree root and leaves into two groups by removing the edge. We assigned a score equal to the number of equal partitions of the two trees (modified from the original algorithm in which the score is twice the number of different partitions, and which deals with un-rooted trees). **Bootstrapping:** In the CCTs we used bootstrapping to assign a measure of reliability to internal edges. Initially, the tree is reconstructed as described above. Then, the set of identifiers was used to create *n* pseudosamples. In each pseudosample, *m* loci (*m* = number of loci in the identifiers) were randomly chosen (with replacement), and a bootstrap tree was built using the same reconstruction method used for the initial reconstruction. Consequently, each internal edge in the reconstructed tree was assigned a number equal to the percentage of bootstrap trees in which the edge exists. **Tree drawing:** reconstructed trees were converted to the Newick tree format using a MATLAB program. Phenograms were created from these strings using the ‘drawgram’ option in the website shown previously.
