# Supplementary material for: Genomic Variability within an Organism Exposes Its Cell Lineage Tree
Source: PLoS Comput Biol. 2005 Oct 28;1(5):e50. doi: 10.1371/journal.pcbi.0010050 (PMC1274291; doi:10.1371/journal.pcbi.0010050)
Supplement: Table S1 — (89 KB DOC) [file pcbi.0010050.st001.doc]

Table S1. List of MS loci used for the CCT model system

| Locus # | Locus name | Repeat unit | Primers (5’-> 3’) |
| --- | --- | --- | --- |
| 1 | 1721 | CT | 1721F = Gggccttcttatattgcttc  1721R = ggaaagactggaccaaagag |
| 2 | 0352 | GT | 0352F = Ctgggaagttcaagtggctgtg  0352R = atcactgcctggaagcgacac |
| 3 | D4S3042 | AC | D4S3042F = AGCTAACTACTCTCCACCCATAC  D4S3042R = CCATGCTAAGTTTATGATGTCTG |
| 4 | 2868 | AC | 2868F = Ttccccaatctcaccgtcactc  2868R = ggcctagcacctcaccttttcc |
| 5 | 8709 | GT | 8709F = Atcatgcagaccctgcctcaag  8709R = actgtggcatctcccctagcag |
| 6 | CSF | AGAT | CSF-F = GGAGCAGAGGAGGTGGCAGAAG  CSF-R = ACACTTGGACAGCATTTCCTGTGTC |
| 7 | 3866 | AAG | 3866F = Ttgccagaacattaagttagaatttgc  3866R = tttgccctctagcatttttcacc |
| 8 | D16S539 | AGAT | D16S539F = ACAAAAAGCCCCAGGGGTTAAGT  D16S539R = ACCATTTAGCGTTTGTGTGTGCAT |
| 9 | 7514 | AAG | 7515F = Tgcgggtcagaaaacaagtctc  7514R = ctgtctagcccattcctgttgac |
| 10 | 9607 | CTT | 9607F = TTTTCACTTGATCCTGCACCAATC  9607R = TTCCACTTTTGCCCAGAGATAAC |
| 11 | D5S2084 | TC/AC | D5S2084F = AGCTGCTTACCACGAATGTC  D5S2084R = TCAGACTTTGAGCCCTGCTA |
| 12 | D12S86 | AC | D12S86F = AGCTAGTCTGGCATGAGCAG  D12S86R = CTATCCCCTGATGATCTCCC |
| 13 | 7959 | GT | 7959F = Gtcagtgctcaggctgaactgc  7959R = tctgcctcctccattccgttac |
| 14 | 8653 | A | 8653F = Cggttacaagctccacagcaatc  8653R = tgacacagagagggaatcctcgtag |
| 15 | 9847 | AC | 9847F = Tgggtcacatcctaccaaccac  9847R = aggccaacaagcaaaatcaagg |
| 16 | 3326 | AAG | 3326F = Ccttttcatgtttgctcgcttg  3326R = cccactcaatcctgatgaatgacc |
| 17 | 0707 | AAG | 0707F = Caccgctggcagttcttttc  0707R = agcaaagacaaggttaagaatcacg |
| 18 | 6458 | AAG | 6458F = Tgagatgggagaagggagaatcac  6458R = ggcgctttccagttactgtttg |
| 19 | 104439 | TTC/CCT | 104439F = Cctggttcttgctatggcaaatg  104439R = ctaatctgctcctcacactacac |
| 20 | 6005 | AC | 6005F = gctggtgcaggaacagttgg  6005R = ggctgctctggctttaagttcc |
| 21 | AC49 | AC | AC49F = CGCACACGGGCAAGAGTAAGAC  AC49R = AGGATTCGGGTGGGGGCAAGG |
| 22 | dxs556 | AC | dxs556F = Gcatccctagatacagtttggc  DXS556R = gccaacttagaaaacagcagggc |
| 23 | AAG30 | AAG | AAG30F = Cacagcctgggagacaagagtg  AAG30R = tgccatcagtagaagcatgagg |
| 24 | 5284 | CTG | 5284F = Gaggctgcagctgctattcaaag  5284R = caatgccattcctgccatctc |
| 25 | 5106 | AC | 5106F = Ggaggtgctcagccatatttgc  5106R = tgacagctctacaccgaattatttgc |
| 26 | 8392 | CTT | 8392F = Gaaagaccagatatggtttgccttc  8392R = agcaatccgggaacacttcatc |
| 27 | 8333 | AGAA | 8333F = Gagccatgttcatgccactg  8333R = caaacccgactaccagcaac |
| 28 | 7381 | AAAGG | 7381F = Ctgggagtcagaagttgcaatg  7381R = gcctcatctcctgtcctgcac |
| 29 | 5994 | AC | 5994F = Gccaaggcaggaagatcacttg  5994R = ccctttcttccacacacccatc |
| 30 | 5802 | AC | 5802F = cccagaccaagaaccaacttgc  5802R = atatggtccgggatgcaaatg |
| 31 | TP53 | AC | TP53f = Agggatattcagcccgaggtg  TP53R = actgccactccttgccccattc |
| 32 | 6424 | AC | 6424F = Aaattgggccagtggtttatcc  6424R = ccactggggtgtgtgtgtgtag |
| 33 | 2068 | GT | 2068F = Gggcagtcgctacaaaggtttc  2068R = tgatggcttctgattacatattccttg |
| 34 | 9804 | AGAGG | 9804F = Gcaacaagagtgaagctccttctcag  9804R = gtccgtgatctgcccgcttc |
| 35 | 7509 | AAG | 7509F = Aattgccattgcaaccacattg  7509R = tggcaccagtgagggtcttattg |
| 36 | AAG44 | AAG | AAG44F = AATCCTTTGAACCCAGGAGG  AAG44R = GAGGGTTTGGTGTGTGTTAGTATTC |
| 37 | 6248 | CTT | 6248F = Agcagcttggatcactggtgtg  6248R = aatctggaggcgaaaattgcag |
| 38 | HEXMUL | AGAGGG | HEXMUL-F = Ccgtctccaccaaaaccagtc  HEXMUL-R = acccaacaccctgctgcttc |
| 39 | 104334 | AAG | 104334F = CTTGAACCCGGGAGGTGGAG  104334R = GGCTCATTAAGGACCTTTTGGG |
| 40 | D21S11 | TCTA/TCTG | D21S11F = ATATGTGAGTCAATTCCCCAAG  D21S11R = TGTATTAGTCAATGTTCTCCAG |
| 41 | BAT40 | A | Bat40F = ATTAACTTCCTACACCACAAC  bat40R = GTAGAGCAAGACCACCTTG |
| 42 | FGA | TTTC/TTCC | FGA-F = GCCCCATAGGTTTTGAACTCA  FGA-R = TGATTTGTCTGTAATTGCCAGC |
| 43 | D8S1179 | TCTA/TCTG | D8S1179F = TTTTTGTATTTCATGTGTACATTCG  D8S1179R = CGTAGCTATAATTAGTTCATTTTCA |
| 44 | D13S317 | TATC | D13S317F = ACAGAAGTCTGGGATGTGGA  D13S317R = GCCCAAAAAGACAGACAGAA |
| 45 | VWA | TCTA/TCTG | VWA-F = CCCTAGTGGATGATAAGAATAATCAGTATG  vwa-r = GGACAGATGATAAATACATAGGATGGATGG |
| 46 | D7S820 | GATA | D7s820F = TGTCATAGTTTAGAACGAACTAACG  D7s820R = CTGAGGTATCAAAAACTCAGAGG |
| 47 | D5S818 | AGAT | d5s818f = GGGTGATTTTCCTCTTTGGT  d5s818r = TGATTCCAATCATAGCCACA |
| 48 | TPOX | AATG | tpox-f = CACTAGCACCCAGAACCGTC  tpox-r = CCTTGTCAGCGTTTATTTGCC |
| 49 | TH01 | AATG | TH01f = GTGGGCTGAAAAGCTCCCGATTAT  TH01r = ATTCAAAGGGTATCTGGGCTCTGG |
| 50 | D3S1358 | TCTA/TCTG | D3s1358f = ACTGCAGTCCAATCTGGGT  d3s1358r = ATGAAATCAACAGAGGCTTG |
| 51 | 7401 | AAG | 7401f = tcacacagctgttaagtggcagag  7401r = catgccctgttccctgctaaag |
