# Supplementary material for: Genomic Variability within an Organism Exposes Its Cell Lineage Tree
Source: PLoS Comput Biol. 2005 Oct 28;1(5):e50. doi: 10.1371/journal.pcbi.0010050 (PMC1274291; doi:10.1371/journal.pcbi.0010050)
Supplement: Table S2 — (88 KB DOC) [file pcbi.0010050.st002.doc]

Table S2a: Cell identifiers for CCT A

Locus Repeat Root A1 A2 A1a A1b A2a A2b A1a1 A1a2 A1b1 A1b2 A2a1 A2a2 A2b1 A2b2

1721 CT 0 0 1 0 0 1 1 -1 1 0 0 1 0 1 1 -1 1 -1 1 0 1 0 -1 1 0 1 0 1 0 1

0352 GT 0 0 -2 1 0 1 -2 1 -3 2 0 2 0 0 -2 2 -2 0 -3 3 -3 2 0 2 0 2 0 0 0 -2

D4S3042 AC 0 0 0 0 0 0 0 0 0 1 0 0 0 0 0 0 0 0 0 1 0 1 0 0 0 0 0 0 0 0

2868 AC 0 0 0 -1 -1 -2 0 -1 0 -1 -1 -2 -1 0 0 -1 0 -1 0 -2 0 -1 -2 -2 -1 -2 -1 -1 -2 0

8709 GT 0 0 0 -1 -1 0 0 -2 0 -2 1 -1 0 1 0 -2 -1 -2 -1 -1 0 -3 2 -2 2 -1 1 1 1 1

CSF AGAT 0 0 0 0 0 -1 0 0 0 0 0 -1 0 -1 0 0 0 0 0 0 0 0 0 -1 0 -1 0 -1 0 -1

3866 AAG 0 0 -1 0 0 0 -1 0 -1 0 0 0 0 0 -1 0 -1 0 -1 0 -1 0 0 0 0 0 0 0 0 0

D16S539 AGAT 0 0 0 0 1 0 0 0 0 0 1 0 1 0 0 0 0 0 0 0 0 0 1 0 1 0 1 0 1 0

7514 AAG 0 0 0 0 0 0 0 0 0 0 0 -1 0 -1 0 0 0 0 0 0 0 0 0 -1 0 -1 0 -1 0 -1

9607 CTT 0 0 0 0 0 0 0 0 0 0 0 0 -1 1 0 0 0 0 0 0 0 0 0 0 0 0 -1 1 -1 0

D5S2084 AC 0 0 0 -2 0 1 0 -2 0 -2 0 0 0 0 0 -2 0 -2 0 -2 0 -2 0 0 0 -1 0 0 0 0

D12S86 AC 0 0 -1 0 1 0 -5 -1 -1 -1 1 0 1 1 -4 0 -3 -1 -1 -1 -1 -1 0 1 1 1 1 1 1 1

7959 GT 0 0 0 0 0 0 -1 0 0 0 0 0 -1 0 -1 0 -1 -1 -1 0 0 0 -2 0 0 0 0 1 -1 0

9847 AC 0 0 -2 1 -2 0 1 1 -2 1 -2 0 -2 0 1 1 1 2 -2 1 -2 1 -2 0 -3 0 -3 0 -2 1

3326 AAG 0 0 0 0 0 0 0 0 1 1 1 0 0 0 0 0 -1 0 1 1 1 1 1 0 1 0 0 0 0 0

0707 AAG 0 0 0 -1 -2 0 0 -1 0 -1 -2 0 -2 0 0 -1 1 -1 0 -1 0 -1 -2 0 -2 0 -2 0 -2 0

6458 AAG 0 0 0 0 0 0 0 0 0 0 0 0 0 0 0 0 0 0 0 0 0 0 0 0 0 0 0 0 0 0

104439 CTT 0 0 0 1 0 -1 0 0 0 1 0 -1 0 -1 1 -1 0 0 0 1 0 1 0 -1 0 0 0 -1 0 -1

6005 AC 0 0 0 0 0 0 0 0 0 0 0 0 0 0 0 0 0 0 0 0 0 0 0 -1 0 0 0 0 0 0

AC49 AC 0 0 0 -1 0 2 0 -1 0 -2 0 1 0 2 0 -1 0 -1 0 -1 0 -1 0 2 0 2 -1 1 0 1

dxs556 AC 0 0 -1 0 0 0 -1 0 -1 0 0 0 0 0 -1 0 -1 0 -1 0 -1 0 0 0 0 0 0 0 -1 0

AAG30 AAG 0 0 0 0 0 1 0 0 0 0 0 1 0 1 0 0 0 0 0 0 0 0 0 1 0 1 0 1 0 1

5284 CTG 0 0 -1 0 0 0 -1 0 -1 0 0 0 0 0 -1 0 -1 0 -1 0 -1 0 0 0 0 0 0 0 0 0

5106 AC 0 0 0 -2 1 -1 0 -2 0 -2 1 0 1 -1 0 -2 0 -2 0 -2 0 -2 1 0 1 -2 1 -1 1 1

8392 CTT 0 0 0 0 0 0 0 0 0 0 0 0 -1 0 0 0 0 0 0 0 0 0 0 0 0 0 -1 0 -1 0

8333 AGAA 0 0 0 0 0 0 1 0 0 0 0 0 0 0 1 0 1 0 0 0 0 0 0 0 0 0 0 0 0 0

7381 AAAGG 0 0 0 0 0 1 0 0 0 0 0 1 0 0 1 0 0 0 0 1 0 0 0 0 0 0 0 0 -1 0

5994 AC 0 0 0 1 0 -1 0 1 0 1 0 -1 0 -1 0 1 0 0 0 1 0 1 0 -1 0 -1 0 -3 0 -1

5802 AC 0 0 0 0 0 0 0 0 0 0 0 0 0 0 0 0 0 0 0 0 0 0 0 0 0 0 0 0 0 0

TP53 AC 0 0 0 0 -1 0 0 0 -1 0 -2 0 -1 0 0 0 0 0 -1 0 -1 0 -2 0 -2 0 0 0 -1 0

6424 AC 0 0 0 2 0 -2 0 2 0 1 1 -1 0 -1 0 2 0 2 0 1 0 2 1 -1 1 -1 0 -2 0 -1

2068 GT 0 0 0 0 0 -1 0 0 0 0 0 -1 0 -1 0 0 0 0 0 0 0 0 0 -1 0 -1 0 -1 0 -1

7509 AAG 0 0 0 0 1 0 0 0 0 0 1 0 -1 0 0 0 0 0 0 0 0 0 2 0 2 0 -1 0 -1 0

AAG44 AAG 0 0 0 0 0 1 0 0 0 -1 0 1 0 1 0 0 0 0 0 -1 0 -1 0 1 0 1 0 1 0 1

6248 CTT 0 0 0 -1 0 0 0 -2 0 -1 0 0 0 0 0 -2 0 -2 0 -1 0 -1 0 0 0 0 0 0 0 0

HEXMUL AGAGGG 0 0 0 0 0 0 0 0 0 0 0 0 0 0 0 0 0 0 0 0 0 0 0 0 0 0 0 0 0 0

104334 AAG 0 0 0 0 -2 0 0 0 0 0 -2 0 -4 0 0 0 0 0 0 0 0 -1 -2 0 -2 0 -4 0 -4 0

X = No signal

Number of amplified loci = 37

**Table S2b: Cell identifiers for CCT B**

Locus Repeat Root B1 B2 B1a B1b B2a B2b B1a1 B1a2 B1b1 B1b2 B2a1 B2a2 B2b1 B2b2

1721 CT 0 0 0 0 -1 0 0 0 0 0 -1 0 -1 1 0 0 0 3 -1 1 0 0 -1 1 0 -1 -1 1 -1 1

0352 GT 0 0 3 -2 0 2 0 0 -1 -2 0 2 3 -3 0 0 0 0 -2 -3 -1 -3 0 3 0 2 3 X -1 -3

D4S3042 AC 0 0 0 0 0 0 0 0 0 0 0 0 0 0 0 0 0 1 0 0 0 0 0 0 0 0 0 0 0 0

2868 AC 0 0 0 0 1 1 0 0 -2 1 1 0 1 2 0 -2 0 1 -2 1 -2 3 0 0 1 -1 1 X 1 1

8709 GT 0 0 0 X -1 -1 0 2 1 0 -1 -1 -2 -1 -1 2 -1 2 1 0 0 0 -1 -2 -2 -2 -2 X -2 -1

CSF AGAT 0 0 -2 0 0 0 0 -1 0 0 0 0 -2 0 0 -1 0 -1 0 1 0 0 0 0 0 0 -2 0 0 0

3866 AAG 0 0 X X -3 -1 -2 0 0 -1 -3 0 0 0 -2 -3 -2 -1 -2 0 0 -1 -3 0 0 0 0 0 0 0

D16S539 AGAT 0 0 0 0 0 0 0 0 0 0 0 0 0 0 1 0 0 0 0 0 0 0 0 0 0 0 0 0 0 0

7514 AAG 0 0 1 0 0 0 1 0 1 0 0 0 -1 0 1 0 1 0 1 0 1 0 0 1 -1 0 -2 1 0 0

9607 CTT 0 0 0 X 0 0 0 X 0 X 0 X 0 0 0 X 0 0 0 -1 0 0 0 0 0 0 0 0 0 -1

D5S2084 AC 0 -1 X X 0 -1 0 -1 -2 -1 0 -1 0 -1 0 -1 0 -2 -3 -1 -3 -1 0 -2 0 -2 0 -1 0 0

D12S86 AC 1 0 X X X X X X 1 0 -3 -1 -1 -1 1 1 0 0 1 0 1 1 -2 -1 -2 -1 -1 -1 -1 -1

7959 GT -1 -1 -3 -3 X X X X X X 2 2 X X -1 2 -2 1 -1 2 -1 1 -2 -2 -2 2 -1 0 -2 -1

8653 A X X X X X X X X X X X X X X X X X X X X X X X X X X X X X X

9847 AC -1 -1 X X -1 -1 -1 -1 -1 1 -1 -1 0 0 -1 0 -1 0 -1 1 -1 1 -3 -1 -1 -1 0 0 0 0

3326 AAG 0 0 0 0 0 1 -3 0 0 0 0 1 0 1 0 0 0 0 1 0 0 0 0 2 0 1 0 1 0 1

0707 AAG 0 0 0 -1 0 0 -1 -1 0 -1 0 0 0 0 -1 -1 -1 -1 0 -1 0 -1 0 0 0 0 0 0 -1 0

6458 AAG 0 0 0 0 2 -1 1 X 2 0 2 -1 2 -1 2 0 2 0 2 0 2 0 2 -1 2 0 2 -2 2 -1

104439 CTT 0 X -1 X 0 X -1 X -1 X 0 X 0 X 0 -3 0 -3 -2 X -1 X 0 X 0 X 0 X 0 X

6005 AC 0 0 0 0 0 0 X X 0 0 0 0 0 0 0 0 0 0 0 0 0 0 0 0 0 0 0 0 0 0

AC49 AC 0 -2 0 -2 0 -3 0 -3 0 -1 -1 -3 0 -1 0 -3 0 -1 0 -1 -3 -1 -1 -2 -1 -3 0 -2 0 -1

dxs556 AC 0 0 0 0 0 0 0 0 0 0 0 0 0 0 0 0 0 0 0 0 0 0 0 0 0 0 0 0 0 0

AAG30 AAG 0 0 0 0 0 0 0 0 0 0 0 0 0 0 0 0 0 3 0 0 0 1 0 0 0 0 0 0 0 0

5284 CTG 0 0 0 -3 0 -3 0 0 0 0 0 0 0 -1 0 0 0 3 0 -3 0 0 0 0 0 0 0 -1 -1 -1

5106 AC 1 -1 -1 0 1 -1 1 1 1 0 1 -1 1 -1 1 2 1 0 1 0 -1 0 0 -1 1 -1 1 -1 1 -1

8392 CTT 0 0 0 0 -1 0 0 0 0 0 -1 0 -1 0 0 0 0 -2 0 0 0 0 -1 0 -1 0 -2 0 -1 0

8333 AGAA 0 0 0 0 -1 0 0 0 0 0 -1 0 -1 0 0 0 0 0 -1 0 0 0 -1 0 -1 0 -1 0 -1 0

7381 AAAGG 0 0 0 0 0 0 0 0 0 0 0 0 0 0 0 0 0 0 0 0 0 0 0 0 0 0 0 -1 0 0

5994 AC 0 0 -1 0 0 0 -1 0 -1 1 0 -3 0 2 -1 0 -1 0 -1 0 -1 X 0 -3 0 -3 0 2 0 1

5802 AC X X X X X X X X X X X X X X X X X X X X X X X X X X X X X X

TP53 AC 0 0 0 0 0 0 0 0 0 0 0 0 0 0 0 0 0 0 0 1 0 0 0 0 0 0 0 -2 0 -1

6424 AC 0 0 -2 -1 -1 -1 -2 0 0 -1 -1 -1 -2 0 -2 -2 -2 -1 0 0 0 -1 0 0 -1 0 -2 -2 -2 -2

2068 GT 0 0 0 1 0 0 0 0 -1 1 0 0 0 -1 0 0 0 0 -1 1 -1 0 0 0 0 0 0 -1 0 -1

9804 AGAGG X X X X X X X X X X X X X X X X X X X X X X X X X X X X X X

7509 AAG 0 0 0 0 0 0 0 0 0 0 0 0 0 0 0 0 0 0 0 0 0 0 0 0 0 0 0 0 0 0

AAG44 AAG 0 0 0 0 0 0 0 0 0 -1 0 0 0 -1 0 0 0 0 0 0 0 -1 0 0 0 -1 0 -1 0 -2

6248 CTT 0 0 0 0 0 0 0 0 0 0 0 1 0 0 0 -1 0 0 0 0 0 0 0 1 0 1 X X 0 0

HEXMUL AGAGGG X X X X X X X X X X X X X X X X X X X X X X X X X X X X X X

104334 AAG 0 0 0 -1 -1 0 0 -1 0 -1 0 0 -1 0 0 -1 0 -1 -1 -1 0 -1 0 0 0 0 -1 0 1 0

D21S11 TCTA 0 0 0 0 X X 0 0 0 0 0 0 0 0 0 0 X X X X -2 X 0 0 0 0 1 0 0 0

BAT40 A 0 0 0 -1 X -3 -1 -1 0 -2 0 1 -1 -1 -1 X -3 X X X -3 X 0 1 0 3 0 0 0 -1

FGA CTTT 0 0 0 0 X X 0 0 0 0 0 0 0 0 0 0 X X X X X X 0 0 0 0 0 0 0 0

D8S1179 TCTA 0 0 0 0 X X 0 0 0 0 0 0 0 0 X X X X X X X X 0 0 0 0 0 0 0 0

D13S317 TATC 0 0 0 0 X X 0 0 0 0 0 0 0 1 0 0 -2 -2 X X -2 -2 0 0 0 0 0 1 0 1

VWA TCTA 0 0 0 0 2 0 0 0 0 0 0 1 0 0 0 0 2 0 X X -2 1 0 1 0 1 0 0 0 0

D7S820 GATA 0 0 0 0 X X 0 0 0 0 0 0 0 0 0 0 0 0 -1 0 0 0 0 0 0 0 0 1 0 0

D5S818 AGAT 0 0 0 0 X X 0 0 0 0 0 0 0 0 0 0 0 0 0 0 X 0 0 0 0 0 0 0 0 0

TPOX AATG 0 0 0 0 X X 0 0 0 0 0 0 0 0 0 0 0 0 0 0 0 0 0 0 0 0 0 0 0 0

TH01 AATG 0 0 0 0 X X 0 0 0 0 0 0 0 0 0 0 0 0 0 0 0 0 0 0 0 0 0 0 0 0

D3S1358 TCTA 0 0 0 0 X X 0 0 0 0 0 0 0 0 0 0 0 0 0 0 0 0 0 0 0 0 0 0 0 0

Locus Repeat B1a1a B1a1b B1b1a B1b1b B2a2a

1721 CT 0 0 0 0 -1 1 -1 1 0 -1

0352 GT 0 0 0 0 -2 -2 -2 -3 2 2

D4S3042 AC 0 0 0 0 0 0 0 0 0 0

2868 AC 0 -2 0 -2 -1 1 -2 -3 1 -1

8709 GT -1 2 -1 1 0 0 1 1 -2 X

CSF AGAT 0 -1 0 -1 0 1 0 1 0 0

3866 AAG 0 -1 -2 -1 1 0 1 0 1 0

D16S539 AGAT 0 0 0 0 0 0 0 0 0 0

7514 AAG 1 -1 1 0 1 -1 0 0 -1 -1

9607 CTT 0 0 0 -1 0 -1 0 1 0 0

D5S2084 AC 0 -1 0 -1 -3 -1 -3 -1 0 -2

D12S86 AC 2 2 1 1 1 0 0 0 -3 -1

7959 GT -2 3 -2 -1 -1 3 -1 2 -2 1

8653 A X X X X X X X X X X

9847 AC -1 0 -1 0 -1 1 -1 -1 -1 -1

3326 AAG 0 0 0 0 1 0 1 0 0 1

0707 AAG -1 -1 -1 -1 -1 -1 0 -1 0 1

6458 AAG 2 0 2 0 2 0 2 1 2 -1

104439 CTT 0 -3 0 X -3 X -2 X 1 X

6005 AC 0 0 0 0 0 0 0 0 0 0

AC49 AC 0 -3 -1 -2 0 -1 0 -1 -1 -3

dxs556 AC 0 0 0 0 0 0 0 0 0 0

AAG30 AAG 0 0 0 0 0 0 0 0 0 0

5284 CTG 0 0 0 -3 0 0 -2 -3 0 0

5106 AC 1 1 1 2 1 0 -1 0 1 -1

8392 CTT 0 0 1 0 0 0 0 0 -1 0

8333 AGAA 0 0 0 0 -1 0 -1 0 -1 0

7381 AAAGG 0 0 0 0 0 0 0 0 0 0

5994 AC -1 0 -1 0 -1 0 -1 1 0 X

5802 AC X X X X X X X X X X

TP53 AC 0 0 0 0 0 1 0 1 0 0

6424 AC -2 -1 -1 -1 1 0 1 1 -1 0

2068 GT 0 0 0 0 -1 1 -1 1 0 0

9804 AGAGG X X X X X X X X X X

7509 AAG 0 0 0 0 0 0 0 0 0 0

AAG44 AAG 0 0 0 -1 0 0 0 0 0 -1

6248 CTT 0 -1 0 X 0 X 0 X X X

HEXMUL AGAGGG X X X X X X X X X X

104334 AAG 0 0 0 -1 2 -1 -1 -1 0 0

D21S11 TCTA 0 0 0 0 0 0 X X X X

BAT40 A -1 -1 -1 1 0 -1 X 3 -3 X

FGA CTTT 0 0 0 0 0 0 0 0 X X

D8S1179 TCTA 0 0 0 0 0 0 X X X X

D13S317 TATC 0 0 0 0 0 0 X X X X

VWA TCTA 0 0 0 0 0 0 0 0 -1 -3

D7S820 GATA 0 0 0 0 -1 0 -1 0 0 0

D5S818 AGAT 0 0 0 0 0 0 0 0 -1 0

TPOX AATG 0 0 0 0 0 0 0 0 0 0

TH01 AATG 0 0 0 0 0 0 0 0 0 0

D3S1358 TCTA 0 0 0 -1 0 0 0 0 0 0

X = No signal

Number of amplified loci = 46

**Table S2c: Cell identifiers for CCT C**

Locus Repeat Root C1 C2 C1a C1b C2a C2b C1a1 C1a2 C1b1 C1b2 C2a1 C2a2 C1a1a C1a2a

1721 CT 0 0 0 X -1 -1 0 0 0 0 -1 -1 -1 -3 0 1 0 0 0 0 0 1 -1 -1 -1 0 0 0 0 0

0352 GT 0 0 X X 0 -1 1 1 0 -1 1 0 0 -1 2 0 1 0 1 1 -1 1 0 -4 0 -3 2 -1 1 -1

D4S3042 AC 0 0 0 0 0 0 0 -1 0 0 0 0 0 0 0 -1 0 -1 0 0 0 0 0 0 0 0 0 -1 0 -1

2868 AC 0 0 X X 0 -1 0 -1 0 2 -1 -3 0 -1 -1 0 2 -1 0 2 0 2 -1 -2 -1 -3 -1 0 2 0

8709 GT 0 0 X X 0 -2 X 0 1 -1 -1 -3 0 -2 1 0 0 0 1 -1 3 -1 0 -3 -1 -3 1 -2 0 -1

CSF AGAT 0 0 0 0 0 0 0 0 0 0 0 0 0 0 0 0 0 0 0 0 0 0 0 0 0 0 0 1 0 0

3866 AAG 0 0 0 0 0 0 0 0 0 0 0 0 0 0 0 0 0 0 0 0 0 0 0 0 0 0 0 0 0 0

D16S539 AGAT 0 0 0 0 0 0 0 0 0 1 0 0 0 0 0 0 1 0 0 1 1 0 0 1 0 0 0 0 0 0

7514 AAG 0 0 0 0 0 0 0 0 0 0 0 0 0 0 0 0 0 0 0 0 0 0 0 1 0 0 0 0 0 1

9607 CTT 0 0 0 1 0 0 0 1 0 2 0 -1 -1 0 0 1 0 1 0 2 0 2 0 -2 0 1 0 1 0 1

D5S2084 AC 0 0 0 0 0 -1 X X X X 0 -1 0 -1 X X X X X X X X X X 0 -2 X X X X

D12S86 AC 0 0 X X 0 0 0 0 -1 -1 0 -1 0 0 -3 0 -1 0 -1 -1 -2 -1 0 -1 -1 -1 -2 -1 -1 0

7959 GT 0 0 X X X X X X X X -1 0 -1 0 -1 0 -1 0 0 0 0 0 -1 0 -1 0 -1 0 -1 0

9847 AC 0 0 1 -1 X X -2 -1 1 -1 -1 -1 -1 -1 -4 -1 -1 -1 1 -1* 2 -1 -1 -1 -1 -1 -5 -1 -2 -1

3326 AAG 0 0 0 -1 0 0 0 -1 0 -1 0 0 0 0 0 -1 0 -2 0 -1 0 -1 0 0 0 0 0 -1 0 -2

0707 AAG 0 0 0 0 0 0 0 0 0 0 0 0 0 0 0 0 0 0 0 0 0 0 0 0 0 0 0 0 0 0

6458 AAG 0 0 0 0 0 0 0 1 0 0 0 0 0 0 0 1 0 1 0 -1 0 0 0 0 0 2 0 1 0 1

104439 CTT 0 0 0 0 0 0 0 0 0 0 0 0 0 0 0 0 -1 0 0 0 0 0 0 0 0 0 -2 0 -1 0

6005 AC 0 0 0 0 0 0 0 0 0 0 0 0 0 0 0 0 0 0 0 0 0 0 0 0 0 -1 0 0 0 0

AC49 AC 0 0 0 0 0 -1 0 2 0 0 0 -1 0 -1 0 2 0 3 0 0 0 0 0 -2 0 0 0 0 0 1

dxs556 AC 0 0 0 0 0 0 0 0 0 0 0 0 0 0 -1 0 0 0 0 0 -2 0 0 0 0 0 -1 0 0 0

AAG30 AAG 0 0 0 0 0 0 0 0 0 0 0 0 0 0 0 0 0 0 0 0 0 0 0 0 0 0 0 0 0 0

5284 CTG 0 0 X 0 X 0 X 0 X 0 X 0 X 0 X 0 X 0 X 0 X 0 X 0 0 0 X 0 X 0

5106 AC 0 0 0 0 0 0 -1 -2 0 -1 -1 -1 0 0 -1 -2 -1 -2 0 -1 0 -1 -1 -1 -1 -1 -1 -3 -1 -2

8392 CTT 0 0 0 0 0 0 0 0 0 0 0 0 0 0 0 0 0 0 0 0 0 0 0 0 0 0 0 0 -1 0

8333 AGAA 0 0 0 0 0 -2 0 0 0 0 0 -2 0 -2 0 0 0 0 0 0 0 0 0 -2 0 -2 0 0 0 0

7381 AAAGG 0 0 0 0 0 1 0 0 0 0 0 1 0 1 0 0 0 0 0 0 0 0 0 1 0 1 0 0 0 -1

5994 AC 0 0 0 1 0 1 0 1 0 1 0 1 0 1 0 1 0 0 0 1 0 1 0 1 0 -3 0 1 0 0

TP53 AC 0 0 0 0 0 0 0 1 0 1 0 0 0 0 2 -1 1 2 0 1 0 0 0 0 0 -1 1 0 1 3

6424 AC 0 0 -1 1 2 0 -2 0 -2 0 1 -1 2 0 -2 0 -2 1 -4 1 -2 0 1 -1 1 -1 -2 -1 -2 0

2068 GT 0 0 0 0 0 0 0 0 0 0 0 0 0 0 0 0 0 0 0 2 0 0 0 0 0 1 0 0 0 0

7509 AAG 0 0 0 0 0 0 0 0 0 0 0 0 0 0 0 0 0 0 0 0 0 0 0 0 -1 0 0 0 0 0

AAG44 AAG 0 0 0 0 0 0 0 -1 0 0 0 0 0 0 0 -1 0 -1 0 0 0 -1 0 0 0 0 0 -1 0 -1

6248 CTT 0 0 0 -2 0 -1 0 -2 0 -2 0 -1 0 0 0 -3 0 -3 0 -2 0 -2 0 -1 0 -1 0 -3 0 -3

104334 AAG 0 0 -1 0 -1 1 -1 0 0 1 -1 2 -2 1 -1 0 0 0 -1 0 0 1 0 2 -2 2 -1 0 0 0

D21S11 TCTA 0 0 X X 1 0 0 0 0 0 1 0 1 0 0 1 1 0 0 0 0 0 X X 1 0 0 1 0 0

BAT40 A 0 0 0 -2 0 -1 0 -2 0 0 0 0 0 0 0 -2 0 -2 0 0 0 0 0 2 0 0 0 0 0 0

FGA CTTT 0 0 0 0 0 0 0 0 0 0 0 0 0 0 0 0 0 0 0 0 0 0 0 0 0 0 0 0 0 0

D8S1179 TCTA X X X X X X X X X X X X X X X X X X X X X X X X X X X X X X

D13S317 TATC 0 0 0 0 0 0 0 0 0 0 0 0 0 0 0 0 0 0 0 0 0 0 0 0 0 0 0 0 0 0

VWA TCTA 0 0 0 0 0 0 0 0 0 0 0 0 0 -1 0 0 0 0 0 0 0 0 0 0 0 0 0 0 0 0

D7S820 GATA 0 0 0 0 0 0 0 0 0 0 0 0 0 0 0 0 0 0 0 0 0 0 0 0 0 0 0 0 0 0

D5S818 AGAT X X X X X X X X X X X X X X X X X X X X X X X X X X X X X X

TPOX AATG 0 0 0 0 0 0 0 0 0 0 0 0 0 0 -1 0 0 0 0 0 0 0 0 0 0 0 -1 0 0 0

TH01 AATG 0 0 0 0 0 0 0 0 0 0 0 0 0 0 0 0 0 0 0 0 0 0 0 0 0 0 0 0 0 0

D3S1358 TCTA 0 0 0 0 0 0 0 0 0 0 0 0 0 0 0 0 0 0 0 0 0 -1 0 0 0 0 0 0 0 0

7401 AGG 0 0 0 0 0 -1 0 0 0 0 0 -1 0 -1 0 0 0 0 0 0 0 0 0 -1 X X 0 0 0 0

Locus Repeat C1a2b C1b1a C1b1b C1b2a C1b2b C2a1a C2a1b C2a2a C2a2b

1721 CT 0 0 0 -1 0 1 0 1 0 1 -1 -2 0 -1 -1 0 -1 0

0352 GT 1 -3 1 1 2 0 1 0 0 0 0 -3 0 -5 0 -4 0 -8

D4S3042 AC 0 -1 0 0 0 0 0 0 0 0 0 0 0 0 0 0 0 0

2868 AC 2 0 0 3 0 0 0 3 1 3 -1 -2 -1 -2 -1 -1 -1 -1

8709 GT 1 3 1 -1 1 -2 3 -1 3 -1 0 -4 -1 -3 -1 -4 -1 -3

CSF AGAT 0 0 0 0 0 0 0 0 0 0 0 0 0 0 0 0 0 0

3866 AAG 0 0 0 0 0 0 0 0 0 0 0 0 0 0 0 0 0 0

D16S539 AGAT 0 0 0 1 0 1 0 1 0 1 0 1 0 1 0 0 0 0

7514 AAG 0 -2 0 0 0 1 0 0 0 -1 -1 1 0 0 0 0 0 0

9607 CTT 0 1 0 2 1 2 0 2 0 2 0 -2 -1 -2 1 0 0 1

D5S2084 AC X X X X X X X X X X X X X X X X X X

D12S86 AC -2 1 -1 -1 -1 -1 -2 -1 -2 -1 1 0 0 -1 -3 -1 1 -1

7959 GT -1 0 -1 0 0 0 0 0 0 0 -1 0 -1 0 -2 0 -1 0

9847 AC -1 -1 1 -1* 1 -1* 2 -1 2 -1 -1 -1 -1 -1 -1 -1 -1 -1

3326 AAG 0 -2 0 0 0 -1 0 0 0 -1 0 0 0 0 0 0 0 0

0707 AAG 0 0 0 0 0 0 0 0 0 0 0 0 0 0 0 0 0 0

6458 AAG 0 1 0 -1 0 -2 0 0 0 0 0 0 0 0 0 2 0 2

104439 CTT -1 0 0 0 0 0 0 0 0 0 0 0 0 0 0 0 -1 0

6005 AC 0 0 0 0 0 -1 0 0 0 0 0 0 0 0 0 -1 0 -1

AC49 AC 0 4 0 -4 0 0 0 0 0 0 0 -2 0 -2 0 0 -1 0

dxs556 AC 0 0 0 0 1 0 -2 0 -2 0 0 0 0 0 1 0 0 0

AAG30 AAG 0 0 0 0 0 0 0 0 0 0 0 0 0 0 0 0 0 0

5284 CTG X 0 X 0 X 0 X 0 X 0 X 0 X 0 X 0 X 0

5106 AC -1 -4 0 -1 1 -3 0 -2 0 -1 -1 -1 -1 -1 -1 -1 -1 -1

8392 CTT 0 0 0 0 -1 0 0 0 0 0 0 0 0 0 0 0 0 0

8333 AGAA 0 0 0 0 0 0 0 0 0 0 0 -2 0 -2 0 -2 0 -2

7381 AAAGG 0 0 0 0 -1 0 0 0 0 0 0 1 0 1 0 1 0 1

5994 AC 0 0 0 1 0 1 0 1 0 1 0 1 0 1 0 -2 0 -2

TP53 AC 1 1 0 1 0 1 0 1 0 0 0 0 0 0 0 -1 0 -1

6424 AC -2 2 -4 1 -4 1 -2 0 -2 -1 1 -1 1 -1 0 -2 1 -1

2068 GT 0 0 0 3 0 1 0 0 0 0 0 0 0 0 0 1 0 0

7509 AAG X X 0 0 0 0 0 0 0 0 0 0 0 0 -1 0 -1 0

AAG44 AAG 0 -2 0 0 0 0 1 -1 0 -1 0 0 0 0 0 0 0 0

6248 CTT 0 -3 0 -3 0 -2 0 -1 0 -2 0 -1 0 -1 0 -1 0 -2

104334 AAG 0 0 -2 -1 -3 0 0 1 0 1 1 2 0 2 -2 2 -2 2

D21S11 TCTA 1 0 0 0 0 0 0 0 1 0 1 0 1 0 1 0 1 0

BAT40 A 0 0 1 0 0 0 -1 0 0 0 0 1 0 0 0 0 0 0

FGA CTTT 0 0 0 0 0 0 0 0 0 0 0 0 0 0 0 0 0 0

D8S1179 TCTA X X X X X X X X X X X X X X X X X X

D13S317 TATC 0 0 0 0 0 0 0 0 0 0 0 0 0 0 0 0 0 0

VWA TCTA 0 0 0 0 0 0 0 0 0 0 0 0 0 0 0 0 0 0

D7S820 GATA 0 0 0 0 0 0 0 0 0 0 0 0 0 0 0 0 0 0

D5S818 AGAT X X X X X X X X X X X X X X X X X X

TPOX AATG 0 0 0 0 0 0 0 0 0 0 0 0 0 0 0 0 0 0

TH01 AATG 0 0 0 0 0 0 0 0 0 0 0 0 0 0 0 0 0 0

D3S1358 TCTA 0 0 0 0 0 0 0 -1 0 -1 0 0 0 0 0 0 0 0

7401 AGG 0 0 0 0 0 0 0 0 0 0 0 -1 0 -1 -1 -1 0 -1

X = No signal

Number of amplified loci = 45

-1* (locus 9847) = "Out of frame" mutation between -1 and -2
