# Supplementary material for: Genomic Variability within an Organism Exposes Its Cell Lineage Tree
Source: PLoS Comput Biol. 2005 Oct 28;1(5):e50. doi: 10.1371/journal.pcbi.0010050 (PMC1274291; doi:10.1371/journal.pcbi.0010050)
Supplement: Table S4 — (26 KB DOC) [file pcbi.0010050.st004.doc]

Table S4. Estimated number of MS mutations in each cell division (mouse)

| Number of repeats | <9 | 9 | 10 | 11 | 12 | 13 | 14 | 15 | >15 | All MS (>=9) |
| --- | --- | --- | --- | --- | --- | --- | --- | --- | --- | --- |
| Number of alleles in mouse genome | >10^6^ | 695030 | 501168 | 317778 | 239548 | 198166 | 162104 | 137848 | 876748 | 3128390 |
| Mutation rate | 0* | 1.04*10^-6^ | 5.05*10^-6^ | 8.81*10^-6^ | 1.23*10^-5^ | 1.70*10^-5^ | 2.37*10^-5^ | 3.30*10^-5^ | 3.54*10^-5^ |  |
| Expected mutations in each daughter cell | 0 | 0.72 | 2.53 | 2.80 | 2.94 | 3.38 | 3.84 | 4.55 | 31.00 | 51.76 |

* In the published data, no MS mutations were found in this category, possibly because the mutation rate is lower than the experimental detection threshold. Here we make a conservative assumption and set the rate at 0.
