# Supplementary material for: Genomic Variability within an Organism Exposes Its Cell Lineage Tree
Source: PLoS Comput Biol. 2005 Oct 28;1(5):e50. doi: 10.1371/journal.pcbi.0010050 (PMC1274291; doi:10.1371/journal.pcbi.0010050)
Supplement: Table S5 — (27 KB DOC) [file pcbi.0010050.st005.doc]

Table S5. Silent cell divisions in Human.

| Number of repeats | <9 | 9 | 10 | 11 | 12 | 13 | 14 | 15 | >15 | All MS (>8) |
| --- | --- | --- | --- | --- | --- | --- | --- | --- | --- | --- |
| Number of alleles in human genome | >10^6^ | 696034 | 457752 | 288054 | 225074 | 196330 | 173372 | 154158 | 790884 | 2981658 |
| Mutation rate | 0* | 1.04*10^-6^ | 5.05*10^-6^ | 8.81*10^-6^ | 1.23*10^-5^ | 1.70*10^-5^ | 2.37*10^-5^ | 3.30*10^-5^ | 3.54*10^-5^ |  |
| Probability for no mutations in each daughter cell | 1 | 0.48 | 0.10 | 0.08 | 0.06 | 0.04 | 0.02 | 0.01 | 7.15*10^-13^ | 6.15*10^-22^ |

* In the published data, no MS mutations were found in this category, possibly because the mutation rate is lower than the experimental detection threshold. Here we make a conservative assumption and set the rate at 0.
