# Supplementary material for: Genomic Variability within an Organism Exposes Its Cell Lineage Tree
Source: PLoS Comput Biol. 2005 Oct 28;1(5):e50. doi: 10.1371/journal.pcbi.0010050 (PMC1274291; doi:10.1371/journal.pcbi.0010050)
Supplement: Table S6 — (28 KB DOC) [file pcbi.0010050.st006.doc]

Table S6. List of MS loci used for *Robinia pseudoacacia*

| Locus # | Locus name | Repeat unit | Primers (5’-> 3’) |
| --- | --- | --- | --- |
| 1 | Rops15 | CT | Rops15f = ctagatgcccattttcaagaatcc  Rops15r = cgcaactaggggctaaatgagg |
| 2 | Rops16 | CT | Rops16f = aaccctaaaagcctcgttatc  Rops16r = tggcattttttggaagacacc |

Note that the primers used for the Rops15 locus in Robinia are different than those used in [39] and are expected to yield products larger by 101bps than those in [39].
