# Supplementary material for: Genomic Variability within an Organism Exposes Its Cell Lineage Tree
Source: PLoS Comput Biol. 2005 Oct 28;1(5):e50. doi: 10.1371/journal.pcbi.0010050 (PMC1274291; doi:10.1371/journal.pcbi.0010050)
Supplement: Table S7 — (50 KB DOC) [file pcbi.0010050.st007.doc]

Table S7. List of MS loci used for *Arabidopsis thaliana*

| Locus # | Locus name | Repeat unit | Primers (5’-> 3’) |
| --- | --- | --- | --- |
| 1 | At5474 | AC | 5474f= tgtgcagctggcttcaagactc  5474r= gagtgcaattacgttccctggac |
| 2 | At2240 | AAG | 2240f= ccaaccattaagaataaagcaagaacc  2240r= ccatgcatatttgcgggcttac |
| 3 | At8907 | CTT | 8907f = tgttggtcacatgagtgtctctgc  8907r = cgaatccaaagggaaaagatgg |
| 4 | At3506 | CTT | 3506f = catatttgtggtccctggctaatc  3506r = ccaccagtgacgaattccaaaac |
| 5 | At5449 | CTT | 5449f = gctttatccaacctccgatgacc  5449r = gtcttcgcagaccatgttgagg |
| 6 | At7727 | AC | At7727f = tgcacccaagaaatagcatggac  At7727r = cacgaggggagtccctagcag |
| 7 | At8112 | AG | At8112f = tctcccatccgtttgctgaatc  At8112r = ttggtgtgccaaagtcaaacaag |
| 8 | At7035 | AG | At7035f = tgcttcaaatgattcaaatgtctcg  At7035r = ttagattagatcgagtggggaaacc |
| 9 | At7187 | AG | At7187f = aagcgctcttcatctcatcatacc  At7187r = ccacgtggaaatcacctttacctc |
| 10 | At4238 | AAG | 4238f = tttggaattagacgcgaagctg  4238r = tgttgatcgccgtttgataagc |
| 11 | At1495 | AAG | 1495f = gccaagacgcagaagaagagtttg  1495r = cctttttggcctgttgctaacc |
| 12 | At3032 | AAG | At3032f = tctgttgccttttctcattgacattc  At3032r = gatttaggaggggcgagagtcc |
| 13 | At9608 | AAG | At9608f = ctccggatcccaaaccttcag  At9608r = tgggatgacaatgacggagaag |
| 14 | At1810 | CTT | At1810f = tgcgcttcttttgttaatttgcag  At1810r = cccgattttcttgaagcttgctc |
| 15 | At5445 | ATGT | At5445f = acaacgactcaaagaagcagagaag  At5445r = caacaacaaattggagagccacag |
| 16 | At3835 | AG | At3835f = tggaactcaacgtggattgtgg  At3835r = agttcaggcgtttgttgcatcc |
| 17 | At4260 | AG | At4260f = atagctattcctacaaggcattttgc  At4260r = tctctttgcgttttggtatcctg |
| 18 | At6446 | AG | At6446f = ttcgaagaagaagaaagcagaaggag  At6446r = ttatcgcgggccaaaattaacg |
| 19 | At3832 | AG | At3832f = gcctgagtcaactcggccataag  At3832r = tacaacaagtggagcgcgtgag |
| 20 | At1471 | C | At1471f = tcaaccggaaaaggactgatttc  At1471r = gaaactaccccataccgcattcc |
| 21 | At1531 | CTT | At1531f = ggggtcctgtctttttgttcttatc  At1531r = tggtaaattctgagcgtccacaac |
| 22 | At7063 | CTT | At7063f = ttggacctgtcaagtgtcaacaatc  At7063r = cgcgtaacgtagagagaatctcaaac |
| 23 | At6642 | AAG | At6642f = tgaacctccggctctttgagtc  At6642r = ccccttcgttccaaacacttagc |
