# Supplementary material for: Genomic Variability within an Organism Exposes Its Cell Lineage Tree
Source: PLoS Comput Biol. 2005 Oct 28;1(5):e50. doi: 10.1371/journal.pcbi.0010050 (PMC1274291; doi:10.1371/journal.pcbi.0010050)
Supplement: Text S1 — (48 KB DOC) [file pcbi.0010050.sd002.doc]

### Text S1. Lineage analysis at the single cell and tissue levels

The purpose of this section is to show that there is a fundamental difference between lineage relations within a group of single cells, and lineage relations within a group of tissues. While lineage relations within a group of single cells are well defined and can be unambiguously represented by a binary tree, lineage relations within a group of tissues (except for the very special case of discrete cell clones) are undefined and cannot be unambiguously represented by a binary tree.

**The lineage relations within a group of single cells are always well defined and unambiguous**

If we take a group of cells from a specific organism and examine the lineage relationships within that group, we will always be able to represent all of these relations by a single binary tree, whose leaves are the single cells. This binary tree completely represents the lineage relations, and there is no ambiguity – there is only one tree which represents these relations, and this tree represents only such lineage relations. The lineage tree may not always be easy to reconstruct and we may not always be able to do it because of practical limitations; but in theory the tree always exists – this means that if we were able to watch the cell divisions and record them, we would know exactly the shape of the tree.

Let’s look at an example:

In this example we are examining the lineage relations within a group of three cells – blue, green and red. We depict these lineage relations by a reconstructed tree which has three leaves, one for each examined cell, and two internal nodes representing the least common ancestors (LCAs) of the cells. (LCA1 is the least common ancestor of the blue and green cells, while LCA2 is the least common ancestor of both the red and blue and the red and green cells. Notice that there is only one possible tree which correctly represents the lineage relationship within the group, and this tree represents only this type of lineage relationship.

LCA2

LCA1

LCA1

LCA2

The full tree

Reconstructed sample tree

**Single cells**

**The lineage relations within a group of discrete cell clones are also well defined and unambiguous**

Cell clones are groups of cells which are all descendants of a single founder cell. Clones A and B are discrete clones if no cell of clone B is a descendant of the founder cell of clone A. **Discrete cell clones are very hard to detect *in vivo*, but they may be produced *in vitro* for building of artificial trees – for control purposes**. Lineage relations within a group of discrete cell clones are always well defined and unambiguous.

Let’s look at an example:

LCA2

LCA1

LCA1

LCA2

**Discrete cell clones**

Reconstructed sample tree

The full tree

In this example, we are examining the lineage relations within a group of three discrete cell clones, each clone comprised of two cells. Because each clone is discrete, beginning in a single founder cell (depicted as a semi-transparent colored circle – notice that the founder cells are not descendants of each other), we can depict the lineage relations within this group of clones by a binary tree, in an unambiguous manner. Notice that this situation is analogous to single cells, because we are essentially reconstructing the tree of the founder cells of the clones, which are all single cells.

**The lineage relations within a group of heterogeneous tissues are un-defined, may be ambiguous and cannot be represented by a binary tree**

Heterogeneous tissues are groups of cells which are not discrete cell clones. **Almost all tissues in the body are heterogeneous tissues**. There are no widely accepted definitions for lineage relations within a group of heterogeneous tissues. Even if we create special definitions for these relations, we may not benefit much from them, because they cannot be unambiguously represented by a binary tree.

Let’s look at an example:

LCA

LCA

**Heterogeneous tissues**

Ambiguous reconstructed tree

The full tree

Incorrect binary representations

In this example the blue and red tissues are composed of two cells each, and the green tissue is composed of three cells. In an attempt to gain insight into the lineage relations within these tissues, we decide that the lineage relations within the founder cells of the different tissues represent the lineage relationships within the tissues themselves. But this definition does not help us very much because the founder cell of all three tissues happens to be the same cell, the LCA. Therefore, there is no binary tree that correctly represents the lineage relations, and if we draw a non-binary tree which does represent these relations, we end up with a tree which is not very informative, and is also ambiguous because it represents not only our special case, but potentially many other cases as well.

**Labeled binary trees of single cells can faithfully represent lineage relations between heterogeneous tissues**

Lineage relations between heterogeneous tissues can be faithfully represented by labeled binary trees in which each node represents a single cell, which can be associated to its’ tissue of origin by its’ label. For example, in such a tree, all nodes with label A represent tissue A.
