# Supplementary material for: Genomic Variability within an Organism Exposes Its Cell Lineage Tree
Source: PLoS Comput Biol. 2005 Oct 28;1(5):e50. doi: 10.1371/journal.pcbi.0010050 (PMC1274291; doi:10.1371/journal.pcbi.0010050)
Supplement: Text S2 — (165 KB DOC) [file pcbi.0010050.sd003.doc]

### Text S2. Full Arabidopsis results

Previous work using clonal assays has shown that plant tissues which are separated from each other by a small radial angle (on the transverse plane) are more likely to be clonally related than tissues which are separated by a larger radial angle (ref. 43). We wished to show that we can achieve the same result using analysis of MS mutations and therefore we took samples which are positioned at different radial positions. Leaves on secondary inflorescences are ideal for this because the inflorescences develop in a spiral fashion during the plants’ development and they are therefore separated by variable radial angles. We would like to emphasize that all tissue samples taken contained somatic cells but not germ line cells (which reside in reproductive tissues). A total of 22 MS loci were amplified for each of 23 DNA samples in the plant. The size of each Arabidopsis leaf sample was ~25mm2. We estimate each sample contains 100000-500000 cells. For each sample we performed a manual comparison of the amplification products to amplification products from an arbitrary reference sample. This comparison yielded a sample identifier for each sample, in which differences between the sample and the reference sample appear as numbers different than 0 (for example in sample 6 in locus 2240, the number 1 indicates that MS 2240 in locus 6 is larger than the same locus in the reference sample by a single repeat unit). The following table contains all 23 identifiers (each row represents a sample; each column represents a locus):

|  | 8112 | 1810 | 3032 | 5445 | 7727 | 4238 | 5449 | 7187 | 2240 | 8907 | 5474 | 9608 | 1495 | 7035 | 3506 | 3835 | 1471 | 4260 | 6446 | 7063 | 1531 | 3832 |
| --- | --- | --- | --- | --- | --- | --- | --- | --- | --- | --- | --- | --- | --- | --- | --- | --- | --- | --- | --- | --- | --- | --- |
| 1 | NaN | 0 | 0 | 0 | NaN | 0 | 0 | NaN | 0 | 0 | 0 | 0 | 0 | NaN | 0 | 0 | 0 | 0 | -1 | 0 | 0 | 1 |
| 2 | 0 | 0 | 0 | 0 | 0 | 0 | 0 | 0 | 0 | 0 | 0 | 0 | 0 | 1 | 0 | 0 | NaN | 0 | 0 | NaN | NaN | 0 |
| 3 | 0 | 0 | 0 | 0 | 0 | 0 | 0 | 0 | 0 | 0 | 0 | 0 | 0 | 1 | 0 | 0 | NaN | 0 | 0 | NaN | NaN | 0 |
| 4 | 0 | 0 | 0 | 0 | 0 | 0 | 0 | 0 | 0 | 0 | 0 | 0 | 0 | 0 | 0 | 0 | NaN | 0 | 0 | NaN | NaN | NaN |
| 5 | 0 | 0 | 0 | 0 | 0 | 0 | 0 | 0 | 0 | 0 | 0 | 0 | 0 | 0 | 0 | NaN | NaN | 0 | 0 | NaN | NaN | NaN |
| 6 | NaN | 0 | 0 | 0 | 0 | 0 | 0 | 0 | 1 | 0 | 0 | 0 | 0 | 1 | 0 | 0 | NaN | NaN | 0 | NaN | NaN | 1 |
| 7 | 0 | 0 | 0 | 0 | 0 | 0 | 0 | NaN | 0 | 0 | 0 | 0 | 0 | 1 | 0 | 0 | 0 | 0 | 0 | 0 | 0 | 1 |
| 8 | 0 | 0 | 0 | 0 | 0 | 0 | 0 | 0 | 0 | 0 | 0 | 0 | 0 | 0 | 0 | 0 | 0 | 0 | 0 | 0 | 0 | 0 |
| 9 | 0 | 0 | 0 | 0 | 0 | 0 | 0 | 0 | 0 | 0 | 0 | 0 | 0 | 0 | 0 | 0 | NaN | 0 | 0 | 0 | 0 | 0 |
| 10 | 0 | 0 | 0 | 0 | 0 | 0 | 0 | 0 | 0 | 0 | 0 | 0 | 0 | 0 | 0 | 0 | NaN | 0 | 0 | 0 | 0 | 0 |
| 11 | 1 | 0 | 0 | 0 | NaN | NaN | NaN | NaN | NaN | NaN | NaN | NaN | NaN | NaN | NaN | 0 | 0 | 0 | 0 | 0 | 0 | NaN |
| 12 | 1 | -1 | 0 | 0 | 0 | 0 | 0 | 0 | 0 | 0 | 0 | 0 | 0 | 0 | 0 | NaN | NaN | NaN | NaN | NaN | NaN | NaN |
| 13 | 1 | 0 | 0 | 0 | 0 | 0 | 0 | 0 | 0 | 0 | 0 | 0 | 0 | 0 | 0 | 0 | NaN | 0 | 0 | 0 | 0 | 1 |
| 14 | 1 | 0 | 0 | 0 | 0 | 0 | 0 | 0 | 0 | 0 | 0 | 0 | 0 | 0 | 0 | 0 | 0 | 0 | 0 | 0 | 0 | 1 |
| 15 | 1 | 0 | 0 | 0 | 0 | 0 | 0 | 0 | 0 | 0 | 0 | 0 | 0 | 0 | 0 | 0 | -1 | -1 | 0 | 0 | 0 | 0 |
| 16 | 1 | 0 | 0 | 0 | NaN | 0 | NaN | NaN | NaN | NaN | NaN | NaN | NaN | NaN | NaN | 0 | 0 | -1 | 0 | 0 | 0 | 1 |
| 17 | 0 | 0 | 0 | 0 | 0 | 0 | 0 | 0 | 0 | 0 | 0 | 0 | 0 | 0 | 0 | 0 | 0 | -1 | 0 | 0 | 0 | NaN |
| 18 | 0 | 0 | 0 | 0 | 0 | -1 | 0 | 0 | 0 | -1 | 0 | 0 | 0 | -1 | 0 | 0 | NaN | -1 | 0 | 0 | 0 | 0 |
| 19 | 0 | 0 | 0 | 0 | 0 | 0 | 0 | 0 | 1 | 0 | 0 | 0 | 0 | 0 | 0 | 0 | 0 | -1 | 0 | 0 | 0 | 0 |
| 20 | 0 | 0 | 0 | 0 | 0 | 0 | 0 | 0 | 1 | 0 | 0 | 0 | 0 | 0 | 0 | 0 | 0 | -1 | 0 | 0 | 0 | 0 |
| 21 | 0 | 0 | 0 | 0 | 0 | -1 | 0 | 0 | 0 | 0 | 0 | 0 | 0 | -1 | 0 | 0 | 0 | -1 | 0 | 0 | 0 | NaN |
| 22 | 0 | 0 | 0 | 0 | 0 | -1 | 0 | 0 | -1 | 0 | 0 | 0 | 0 | -1 | 0 | 0 | -1 | -1 | 0 | 0 | 0 | NaN |
| 23 | 0 | NaN | NaN | NaN | 0 | -1 | 0 | NaN | 0 | 0 | 0 | 0 | 0 | NaN | 0 | 0 | 0 | -1 | 0 | 0 | 0 | NaN |

Information on somatic mutations in the plant was extracted by comparing the identifiers. For each locus the majority vote value (value shared by largest number of samples) was considered to be the base-line value, and different values were considered mutations. A total of 40 mutations in 17 samples were detected, of which 4 occurred in one sample only, and 36 occurred in two or more samples. The position of each of these shared mutations was marked in a transverse scheme of the plant (Fig. 3c in the main text), and analysis of this scheme revealed that shared mutations tend to be physically clustered. The genetic distance between any two samples was defined as the sum of different numbers in the identifiers of the two samples. The physical-radial distance between any two samples was defined to be one of 4 categories: Samples from the same tertiary inflorescence of the plant were considered to be closest, followed by samples on the same secondary (but not tertiary) inflorescence, samples on adjacent secondary inflorescences, and finally samples on distant secondary inflorescences, which were considered to have the largest physical-radial distance (see Fig. 3b in the main text for examples of tertiary and secondary inflorescences). Comparison of the genetic and physical-radial distances between all pairs of samples revealed a strong correlation. The comparison is shown graphically in the following figure:


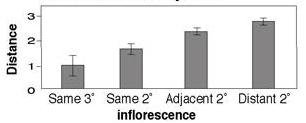


The correlation between genetic and physical-radial distances is statistically significant for most categories, as can be seen in the following table (statistically significant values shaded):

|  | same 3^0^-same 2^0^ | same 3^0^-adjacent 2^0^ | same 3^0^-distant 2^0^ | same 2^0^-adjacent 2^0^ | same 2^0^-distant 2^0^ | adjacent 2^0^-distant 2^0^ |
| --- | --- | --- | --- | --- | --- | --- |
| Correlation (P value) between genetic and physical-radial distances | 0.1417 | 0.0074 | 0.000835 | 0.0080 | 0.00003 | 0.0248 |

**Additional Arabidopsis experiments**

In addition to this plant, experiments were performed on two other plants (plant #11, plant #12). In plant #11, DNA from 36 tissue samples was obtained, and the radial position of each sample was recorded. 23 MS loci were amplified for each DNA sample by PCR. A total of 17 mutations (relative to majority vote) were identified in 9 DNA samples in 6 MS loci. Each sample was assigned an identifier from the 23 amplified loci. Genetic distance between the samples was calculated by using the “equal or not” distance function, which is the sum of amplified loci with a different value between samples.

In plant #12, DNA from 47 tissue samples was obtained, and the radial position of each sample was recorded. 23 MS loci were amplified for each DNA sample by PCR. A total of 19 mutations (relative to majority vote) were identified in 19 DNA samples in a single MS locus. Each sample was assigned an identifier from the 23 amplified loci. Genetic distance between the samples was calculated by using the “equal or not” distance function.

Data from plants #11 and #12 were combined and the genetic distances were plotted (using Excel software) against the radial distances. A positive trend was observed in the plot and a linear fit of the plot produced a coefficient of 0.0006 and a R^2^ value of 0.0036.
