# Supplementary material for: Genomic Variability within an Organism Exposes Its Cell Lineage Tree
Source: PLoS Comput Biol. 2005 Oct 28;1(5):e50. doi: 10.1371/journal.pcbi.0010050 (PMC1274291; doi:10.1371/journal.pcbi.0010050)
Supplement: Text S3 — (A) Reconstructing cell lineage trees from DNA extracted from cell clones. (B) Selection criteria for MSs. (29 KB DOC) [file pcbi.0010050.sd004.doc]

**Text S3. Reconstruction Using Cell Clones and MS Selection Criteria**

**Text S3A. Reconstructing cell lineage trees from DNA extracted from cell clones.** We approximate the identifier of a cell C by analyzing a cell clone L of it (this is a current limitation of our procedure, not only in the context of CCTs). Because mutations are expected to occur during proliferation from a founder cell to its clone, a heterogeneous sample is generated. Let M be an MS allele with a probability p for a mutation (per cell division). Our convention for approximating the relative allelic value of C on M is to take the main peak of the Capillary Electrophoresis Histogram (CEH) of L on M. The probability that this indeed gives the correct relative allelic value for M depends on p, on the depth d of the extant tree induced on the cells of the clone L, and on the structure of the extant tree. We elaborate on this below, and also explain why it does not matter if some errors are introduced by this approximation.

One would need the product p*d to be small. The fraction of leaves of the extant tree with no mutation in M is expected to be at least (1-p)^d^, which is approximately (1 - p*d). In the CCTs the values of the parameters are: p < 1/100 and d < 50, hence the fraction of leaves without mutation is above 50%. Moreover, those leaves that do have mutations do not form one peak (with relative weight p*d), but rather two or more smaller peaks (due to the fact that mutations may have more that one possible outcome). Hence one would expect the main peak of the CEH of L to correspond to the relative allelic value of C.

One could have hoped that “the law of large numbers” would work in our favor. That is, as the number of cells in L increases, the probability of large deviations from the expectation decreases, and that the probability that the main peak corresponds to the relative value of C tends to 1. However, this is incorrect, because of dependency between allelic values of leaves that share portions of their paths to C. The exact probability that the main peak indeed corresponds to the relative value of C depends on the structure of the extant tree, which is unknown to us. However, under the assumption that the cells of the clone are random cells of a cumulative tree of depth d, it can be shown that this probability is at least 1-3p. (Essentially, the main source of error is a mutation in one of the two edges adjacent to the root, and the secondary source is mutations in two of the four edges at distance 2 from the root.)

Hence the probability that the main peak of L differs from the relative value of C can be estimated as 3p (or less, under favorable structures for the extant tree, or somewhat more, under unfavorable structures). This roughly corresponds to viewing the identifier obtained by considering the main peaks of L as if it is an identifier of a random hypothetical descendent cell (that we shall name C’) of C, at depth 3 (rather than depth d which is the depth of the cells of the clone).

An important point to notice is that for the CCT reconstruction problem, it does not matter much whether we are working with the identifier of C’ or of C. If C is a leaf of the tissue culture tree, then C’ is simply a somewhat deeper leaf. The condensed tree for all the leaves of C’ is identical to that of the condensed tree for the C leaves. Reconstructing the condensed tree based on the C’ identifiers rather than the C identifiers is slightly more difficult, because in the extant trees, the branch lengths for the C’ tree are somewhat larger than the corresponding branch lengths for the C tree, and hence there is higher probability for colliding mutations.

In some of our tree reconstruction algorithms we also used the identifier of the root. Two comments are in order here.

1. When C is not a leaf of the tissue culture tree but rather its root, then the corresponding C’ can be though of as if the root was three levels further away from the leaves. This again does not offer additional conceptual difficulties.

2. In cases when we do not have the identifier for the root (e.g., a new born baby), but wish to run a reconstruction algorithm that takes as input also an identifier for the root, one can construct a “virtual root” by for each MS allele, taking the most common allelic value over all cells as the value for the virtual root. This is especially justified if mutations can be thought of as symmetric (same probability for +1 and -1).

**Text S3B. Selection Criteria for Microsatellites.**

We wrote a computer program to search for MS loci in any sequenced genome, and created a database of all MS in Human, Mouse and other organisms. This allows quickly choosing any MS and designing primers for it. Selection of MS loci is based on two major criteria. One is a desirable mutation rate. Although it isn't possible to know this a priori, as there are several parameters affecting the rate, a few rules of thumb enable making a pretty good guess: (i) for a given repeat unit, the mutation rate increases with an increasing number of repeats (e.g. AC30 will usually have a higher mutation rate than AC20); (ii) in general, loci with shorter repeat units mutate faster than loci with longer repeat units Another criterion is the amount of PCR stutter which is usually higher for shorter repeat unites and for higher number of repeats. In general, we avoided choosing mono-repeats (with a single exception) in order to reduce the stutter to a level that will enable signal analysis. For the current work we have intentionally selected a wide variety of basic unit lengths and number of repeat in order to learn more about the somatic mutations of MS.
