# Supplementary material for: Genomic Variability within an Organism Exposes Its Cell Lineage Tree
Source: PLoS Comput Biol. 2005 Oct 28;1(5):e50. doi: 10.1371/journal.pcbi.0010050 (PMC1274291; doi:10.1371/journal.pcbi.0010050)
Supplement: Text S4 — (27 KB DOC) [file pcbi.0010050.sd005.doc]

**Text S4. Ignoring the effect of allelic crossovers.** The CEH obtained from amplifying an MS locus from a cell will usually yield two alleles peaks. When comparing the two allelic peaks of two different cells C1 and C2, there is a certain probability that a crossover event occurred between the two cells (meaning that the lower allelic peak of C1 and the higher allelic peak of C2 correspond to the same allele, and vice versa). When reconstructing CCTs, we make the assumption that no crossover occurred. This simplifies the reconstruction algorithm. More sophisticated reconstruction algorithms may take into account the possibility of crossover, get somewhat higher probability of correct reconstruction at a cost of using a more complicated and time consuming algorithm. We chose not to do so at this point.

We present mathematical analysis that addresses the following question: given a possibly ambiguous situation, what is the probability that we make an error? The analysis we perform supports our assumption that crossover events are highly unlikely. For simplicity, we assume the step-wise mutation model. Most current models of the evolutionary dynamics of microsatellites are derived from the stepwise mutation model [31]. Preliminary analysis of mutations in the tissue culture model system (data shown in Supplementary Data 2) also supports this assumption (data not shown).

Assume the allelic peaks of C1 are at s1 and h1 (with s1 < h1) and the allelic peaks of C2 are at s2 and h2 (with s2 < h2). Assume that the probability of mutation in a single cell division is p, and that the length of the path joining C1 and C2 (going through their least common ancestor) is d. We assume that p*d is significantly smaller than 1 so that mutations are rare.

Observe that b = |s1 - s2| + |h1 - h2| mutations suffice in order to explain the data if no crossover occurs, and c = |s1 - h2| + |h1 - s2| mutations are required in order to explain the data if a crossover did occur. Let t = min(h1,h2) - max(s1,s2). It can be seen that unless t is positive, b = c, and then the event of no crossover is only mildly more likely to explain the data as the event of a crossover. (In both cases, the number of mutations is the same, but the distribution of which allele got the mutations is less balanced, and hence less likely, if a crossover occurred.) When t is positive, 2t more mutations are required by a crossover event than a no crossover event. The ratio between the probabilities of these two events is in the order of (pd)^2^, making the crossover choice unlikely when pd is much smaller than one.

Hence the question of how often crossovers contaminate the data depends on the typical value of t (further analysis is required regarding the observed distribution of t). An important point to add here is that if s1=h1 (or s2=h2) then crossover cannot contaminate the comparison between the two cells, so these cases may be counted as if t is infinite.

Moreover, the situation may be in fact better than the analysis suggests, because the ambiguous situations may rarely arise (e.g., if in the root cell, the two allelic peaks are far apart).
